# Complex Interaction Networks Among Cyanolichens of a Tropical Biodiversity Hotspot

**DOI:** 10.3389/fmicb.2021.672333

**Published:** 2021-06-04

**Authors:** Ulla Kaasalainen, Veera Tuovinen, Geoffrey Mwachala, Petri Pellikka, Jouko Rikkinen

**Affiliations:** ^1^Department of Geobiology, University of Göttingen, Göttingen, Germany; ^2^Finnish Museum of Natural History, University of Helsinki, Helsinki, Finland; ^3^Department of Ecology and Genetics, Uppsala University, Uppsala, Sweden; ^4^East African Herbarium, National Museums of Kenya, Nairobi, Kenya; ^5^Department of Geosciences and Geography, University of Helsinki, Helsinki, Finland; ^6^State Key Laboratory of Information Engineering in Surveying, Mapping and Remote Sensing, Wuhan University, Wuhan, China; ^7^Organismal and Evolutionary Biology Research Programme, Faculty of Biological and Environmental Sciences, University of Helsinki, Helsinki, Finland

**Keywords:** lichen, symbiosis, mycobiont, photobiont, photobiont-mediated guild, peltigerales, *Nostoc*

## Abstract

Interactions within lichen communities include, in addition to close mutualistic associations between the main partners of specific lichen symbioses, also more elusive relationships between members of a wider symbiotic community. Here, we analyze association patterns of cyanolichen symbionts in the tropical montane forests of Taita Hills, southern Kenya, which is part of the Eastern Afromontane biodiversity hotspot. The cyanolichen specimens analyzed represent 74 mycobiont taxa within the order Peltigerales (Ascomycota), associating with 115 different variants of the photobionts genus *Nostoc* (Cyanobacteria). Our analysis demonstrates wide sharing of photobionts and reveals the presence of several photobiont-mediated lichen guilds. Over half of all mycobionts share photobionts with other fungal species, often from different genera or even families, while some others are strict specialists and exclusively associate with a single photobiont variant. The most extensive symbiont network involves 24 different fungal species from five genera associating with 38 *Nostoc* photobionts. The *Nostoc* photobionts belong to two main groups, the *Nephroma*-type *Nostoc* and the *Collema*/*Peltigera*-type *Nostoc*, and nearly all mycobionts associate only with variants of one group. Among the mycobionts, species that produce cephalodia and those without symbiotic propagules tend to be most promiscuous in photobiont choice. The extent of photobiont sharing and the structure of interaction networks differ dramatically between the two major photobiont-mediated guilds, being both more prevalent and nested among *Nephroma* guild fungi and more compartmentalized among *Peltigera* guild fungi. This presumably reflects differences in the ecological characteristics and/or requirements of the two main groups of photobionts. The same two groups of *Nostoc* have previously been identified from many lichens in various lichen-rich ecosystems in different parts of the world, indicating that photobiont sharing between fungal species is an integral part of lichen ecology globally. In many cases, symbiotically dispersing lichens can facilitate the dispersal of sexually reproducing species, promoting establishment and adaptation into new and marginal habitats and thus driving evolutionary diversification.

## Introduction

**FIGURE 1:**
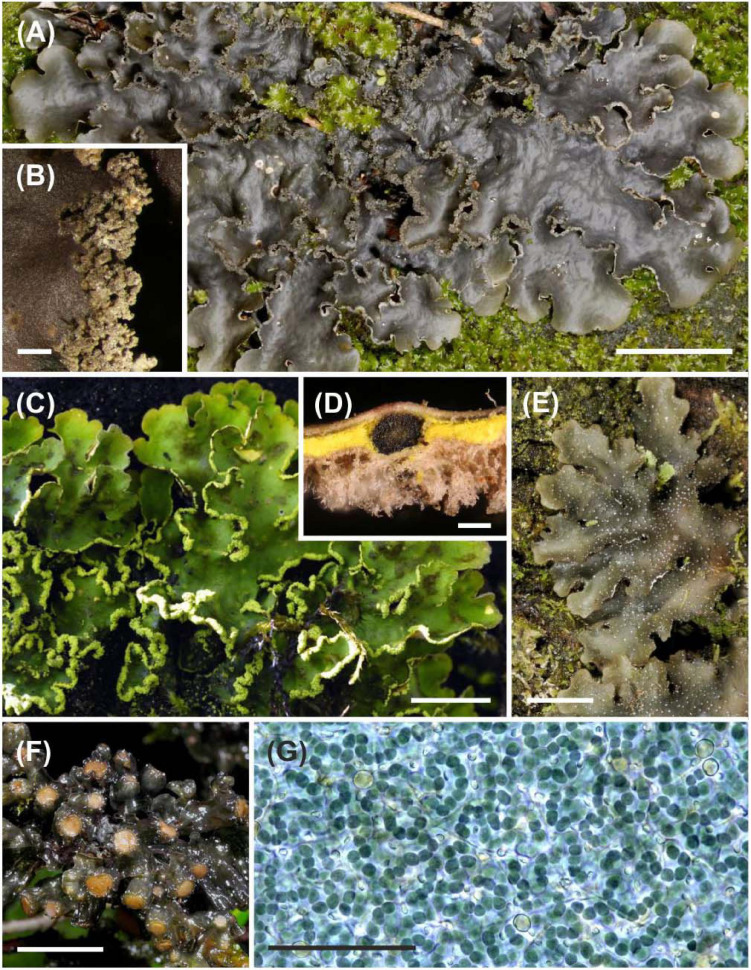
Structural diversity of cyanolichens in the Taita Hills, Kenya. **(A)** Common bipartite cyanolichen *Sticta sublimbata*. **(B)** *Sticta sublimbata* produces coralloid aggregates of soredia (symbiotic propagules containing *Nostoc* cyanobionts) on thallus margins. **(C)** Cephalodiate lichen *Crocodia aurata* with green algal photobiont layer and powdery soredia (symbiotic propagules containing also green algal *Dictyochloropsis* photobionts) on thallus margins. **(D)** *Nostoc* cyanobionts of *Crocodia aurata* are housed in cephalodia within the yellow medulla of the thallus. **(E)** *Pseudocyphellaria argyracea*, another relatively common bipartite cyanolichen species. **(F)** The mycobiont of the bipartite cyanolichen *Leptogium javanicum* produces sexual ascospores in abundant apothecia. **(G)** *Nostoc* cyanobiont of *Leptogium* OTU K14, the large hyaline cells are nitrogen-fixing heterocysts. Scale bars, 1 cm in **(A)**; 0.5 mm in **(B)**; 5 mm in **(C,E,F)**; 0.1 mm in **(D)**; and 50 μm in **(G)**.

Lichens are highly successful self-sustaining ecosystems involving lichen-forming fungi and phototrophic algae and/or cyanobacteria (Hawksworth and Grube, 2020). In the so-called bipartite lichens (Figures 1A,E), the main symbiotic association involves one primary fungus (mycobiont) and photosynthetic algae or cyanobacteria (photobionts). If the lichen mycobiont associates with both algae and cyanobacteria (Figure 1C), the latter are usually housed in specialized structures named cephalodia (Figures 1D,G). A certain degree of symbiont specificity is a prerequisite for long-lasting symbiotic relationships. Within Lecanoromycetes (Ascomycota), the largest class of lichen-symbiotic fungi, most mycobionts seem to be highly specific in their choice of photobionts (Dahlkild et al., 2001; Otálora et al., 2010; Dal Grande et al., 2014; Nyati et al., 2014; Leavitt et al., 2015; Chagnon et al., 2018; Jüriado et al., 2019; Dal Forno et al., 2020; Lindgren et al., 2020). During dispersal, the symbiotic partners can either disperse together within symbiotic propagules (Figure 1B) (vertical transmission), or the symbionts reproduce separately; for example, the mycobionts may produce ascospores in apothecia (Figure 1F) and re-establish the symbiotic association after dispersal (horizontal transmission). While vertical transmission helps to maintain established associations between compatible partners, horizontal transmission can promote the establishment of novel types of associations but also poses the risk of failure if compatible symbionts are not encountered (Bright and Bulgheresi, 2010). Many lichens, including some species in most genera of cyanolichens (lichens with cyanobacterial photobionts), utilize both means of dispersal and photobiont transmission.

Ecological guilds refer to groups of taxa that exploit the same resource, regardless of their taxonomic relationships. Among lichen-symbiotic fungi, several different species can often depend on the same specific type of photobiont and form mutually interacting communities that are called photobiont-mediated lichen guilds (Rikkinen, 2003). As many mycobionts only produce fungal spores, they must reestablish their association with a compatible photobiont at each reproductive cycle. Compatible photobionts can potentially be obtained from two main sources, either from the environment through the recruitment of free-living photobionts, if such exist, or from the pool of lichenized photobionts, maintained by previously established lichens. Shared symbiont specificity may lead to different types of facilitative interactions between different lichens, for example, because some spore-dispersing mycobionts require the prior “seeding” of appropriate photobionts by other lichens that effectively propagate and distribute the appropriate photobiont within their symbiotic propagules (Rikkinen et al., 2002; Fedrowitz et al., 2011, 2012; Dal Grande et al., 2014; Belinchón et al., 2015), or even by bryophytes that house appropriate cyanobacteria (Cornejo and Scheidegger, 2016; Zúñiga et al., 2017).

The large majority of fungi of the Peltigerales (Lecanoromycetes) establish lichen symbioses with *Nostoc* cyanobionts (Nostocales, Cyanobacteria). There are many general differences between prokaryotes and eukaryotes regarding factors that shape species evolution, and the species delimitation among Cyanobacteria remains particularly ambiguous (Dvořák et al., 2015). It has even been proposed that ecological differences may sometimes give a more solid foundation for species delimitation among bacteria than the often enigmatically evolving lineages within ecotypes (Kopac et al., 2014; Dvořák et al., 2015). For example, the *Nostoc* symbionts of most cyanolichens are known to belong to two main groups that consistently associate with different groups of specific hosts and can be conveniently identified on the basis of tRNA^Leu^ (UAA) intron (trnL) sequences as well as through phylogenetic analysis of the 16S rDNA region (Olsson et al., 2012; Kaasalainen et al., 2015). These groups of *Nostoc* cyanobionts are named *Nephroma*-type *Nostoc* and *Collema*/*Peltigera*-type *Nostoc*, after representative genera among the spectra of fungal hosts. Accordingly, the mycobionts associating with the two groups collectively form the *Nephroma* guild and the *Peltigera* guild, respectively.

The underlying processes of symbiont acquisition and the functioning of symbiotic communities remain poorly understood, even though they may obviously have a major impact on the ecology, diversification, and geographical distribution of lichen species (Peksa and Skaloud, 2011; Muggia et al., 2014; Magain et al., 2017; Ertz et al., 2018; Jüriado et al., 2019). Here, we analyze the structure of photobiont-mediated guilds within the rich cyanolichen biota of a diverse tropical environment. We provide a comprehensive view of interactions within one well-known biodiversity hotspot and take the first step in unraveling how regional association patterns in the tropics link to previously recognized patterns of symbiont specificity in cyanolichens.

## Materials and Methods

### Sampling of Biological Material and Amplification of the trnL Marker Region

A total of 393 cyanolichen specimens were sampled (Supplementary Table 1), all collected from montane forests of Taita Hills and Mt. Kasigau in southern Kenya (Supplementary Figure 1), which form the northernmost part of the Eastern Arc Mountains and are renowned for high species richness and many local endemics, including among lichens and associated fungi (Suija et al., 2018; Kaasalainen et al., 2021). The Eastern Arc, together with other eastern African montane regions, including, for example, the Southern and Albertine Rift and the Ethiopian Highlands, form the Eastern Afromontane biodiversity hotspot (Mittermeier et al., 2004). The study locations and vegetation of the area have been described in more detail by Enroth et al. (2013, 2019), Stam et al. (2020), and Kaasalainen et al. (2021).

All the cyanolichens analyzed in this study belong to Peltigerales (Ascomycota) and many of them are common epiphytes in the montane cloud forests of the region. The cyanolichen specimens were identified based on thallus morphology and/or phylogenetic analyses (Swinscow and Krog, 1988; Kaasalainen et al., 2021). The reproductive mode of each lichen specimen was determined under dissecting microscope: A = fungal apothecia present (Figure 1F); S = symbiotic propagules (isidia, phyllidia, or soredia; Figures 1A–C) present.

To assess the genetic identities of the cyanobionts the cyanobacterial trnL was used. DNA was extracted from small lichen thallus fragments using the DNeasy Plant Mini Kit (Giagen AB, Solna, Sweden) or the GeneJET Genomic DNA Purification Kit (Fermentas, Helsinki, Finland) following the manufacturer’s instructions. The trnL was amplified using primers tRNALeu-outF (5′-ggaattcggggrtrtggygraat-3′) and tRNALeu-outR (5′-tcccgggg ryrgrgggactt-3′) and sequenced with tRNALeu-inF (5′-agaatt cggtagacgcwrcggactt-3′), trnL_UFII (5′-ggtagacgctacggactt-3′), and trnL_UR (5′-gggacttgaacccacacgacc-3′) (Paulsrud and Lindblad, 1998; Fedrowitz et al., 2011, 2012). The reactions were performed in a 50-μl volume containing dNTPs at 0.2 mM (Finnzymes, Espoo, Finland), each primer at 0.2 μM, 0.5 mg/ml of BSA, and 0.03 U/μl of DynaZyme II DNA polymerase (Finnzymes, Espoo, Finland). The heating cycle was as follows: the initial denaturation of 2 min at 94°C was followed by 35 cycles of 30 s at 94°C, 30 s at 56°C, and 30 s at 72°C, with a final extension of 10 min at 72°C.

All PCR products were purified using a GeneJET PCR-purification kit (Fermentas, Helsinki, Finland). Sequencing was performed by Macrogen Inc. (Korea/Europe). Sequencing chromatograms were assembled and aligned using BioEdit 7.0.9 (Hall, 1999) and PhyDE-1 v0.997 (Müller et al., 2005). All obtained sequences were deposited in the NCBI GenBank (RRID:SCR_002760) (NCBI Resource Coordinators, 2016) and the accession numbers are listed in Supplementary Table 1.

### Diversity Estimation

The completeness of sampling and the overall fungal OTU/species and cyanobacteria variant richness in the sampled groups were estimated by calculating the Chao 1 richness estimator (Chao, 1984) using EstimateS v9.1.0 (Colwell, 2013). The calculations were done based on one pooled sample of each mycobiont taxon (species or OTUs) and trnL variant abundance data. The estimations were calculated for the following sets of specimens: for all mycobionts, for all *Nephroma* guild mycobionts, and for all *Peltigera* guild mycobionts, respectively, for all cyanobacterial variants, for all *Nephroma*-type *Nostoc*, and for all *Collema*/*Peltigera*-type *Nostoc* variants, respectively. The Chao1 estimator was calculated using the bias-corrected formula for *Nephroma* guild mycobionts, *Peltigera* guild mycobionts, and all cyanobionts, respectively, and the classic formula when estimating the diversity of all mycobionts, *Nephroma*-type *Nostoc*, and *Peltigera*-type *Nostoc*, respectively, as recommended by Colwell (2013).

### Cyanobacterial trnL Sequence Analyses

To depict the diversity among the lichen cyanobionts, the cyanobacterial trnL variants were compiled to networks using the median-joining method of Network (Bandelt et al., 1999). The two main groups of *Nostoc* cyanobionts associating with Peltigeralean mycobionts (*Nephroma*-type *Nostoc* and *Collema*/*Peltigera*-type *Nostoc*) can be unambiguously separated on the basis of trnL sequences (Kaasalainen et al., 2015). However, certain regions of the *Collema*-type and *Peltigera*-type sequences within the *Collema*/*Peltigera*-type *Nostoc* should not be aligned together (Paulsrud and Lindblad, 1998; Kaasalainen et al., 2015), and therefore, three trnL networks were constructed, for the *Nephroma*-type *Nostoc*, *Collema*-type *Nostoc*, and *Peltigera*-type *Nostoc* trnL sequences, respectively. Additionally, the new trnL sequences were compared to existing sequences in the NCBI GenBank with Blast searches (Altschul et al., 1990) (database accessed 28.1.2021).

### Bipartite Interaction Network Analyses

The bipartite interaction network of the mycobiont species/OTUs and cyanobacterial variants was constructed using the R v4 (RRID:SCR_001905) (R Development Core Team, 2011) package “bipartite” v2.15 (Dormann et al., 2008). The package was also used to calculate standardized specialization index d’ describing the degree of interaction specialization for each mycobiont species (Dormann, 2011). Additionally, the following network-level indices were calculated separately for *Nephroma* guild and *Peltigera* guild interaction networks and for mycobiont species and cyanobiont variants: the overall specialization within the network H_2_’, web asymmetry (the difference between the numbers of associating mycobiont taxa and cyanobacterial variants) (Blüthgen et al., 2007), partner diversity (Shannon diversity of the number of interactions for the species of that level), and niche overlap (the similarity of interaction pattern between taxa).

NODF nestedness metric (reflecting the structure of the network) (Almeida-Neto et al., 2008) and the connectance (realized proportion of all possible links) calculations were performed separately for *Nephroma* guild and *Peltigera* guild interaction matrices using NeD (Strona et al., 2014). For the estimation of the statistical significance of the NODF metric, a null model with proportional column and row totals (CE) was used with 100 null matrices.

### Photobiont Diversity and Sharing: Additional Statistical Comparisons

Unpaired *t*-test was used to determine the significance of differences in photobiont diversity (number of cyanobacterial variants per number of specimens) and extent of sharing (standardized specialization index d’) between several different groups of mycobiont taxa (Supplementary Table 2): (a) between bipartite cyanolichens (housing only cyanobacterial photobionts) and cephalodiate lichens (housing both green algal and cyanobacterial symbionts), (b) between fertile lichen species with apothecia and lacking symbiotic propagules and species producing symbiotic propagules but lacking apothecia, and (c) between *Nephroma* and *Peltigera* guild mycobionts. For comparisons (a) and (b), only taxa with at least two specimens were included in the analyses, excluding 23 species and specimens. Additionally, for (b), three species with both apothecia and symbiotic propagules [*Leptogium* OTU E3 (morphotype coralloideum), *Leptogium* OTU K10 (morphotype cyanescens), and *Pannaria* sp. 3], as well as *Peltigera dolichorrhiza* lacking both, were excluded from the comparison. For comparison (c), the three species associating with both *Nephroma*- and *Collema*/*Peltigera*-type cyanobionts [*Crocodia* sp. 2, *Leptogium* OTU K14 (morphotype cochleatum), and *Sticta* sp. 3] were excluded from the analyses. This resulted in the following comparisons for both variables: (a) 48 bipartite cyanolichen species–3 cephalodiate lichen species, (b) 15 fertile lichen species–36 lichen species with only symbiotic propagules, and (c) 19 *Nephroma* guild lichen species–52 *Peltigera* guild lichen species. Additionally, Fisher’s exact test was used to determine the significance of difference in the commonness of photobiont sharing between the *Nephroma* guild and *Peltigera* guild lichen species with the same exclusions as in comparison (c). The significance level was set to 0.05 for all analyses.

## Results

### Diversity of Lichen Mycobionts and Photobionts

The studied 393 cyanolichen specimens represented 74 different cyanolichen species or OTUs, belonging to four families and eight genera of Peltigerales: Collemataceae (seven *Collema* and 40 *Leptogium* spp./OTUs), Lobariaceae (three *Crocodia*, two *Lobaria*, three *Pseudocyphellaria*, and 11 *Sticta* spp.), Pannariaceae (five *Pannaria* spp.), and Peltigeraceae (three *Peltigera* spp.) (Supplementary Table 2). Of the lichen cyanobionts, 156 belonged to the group of *Nephroma*-type *Nostoc* (22 trnL variants) and 237 belonged to the group of *Collema*/*Peltigera*-type *Nostoc* (94 trnL variants) (Supplementary Figure 2).

Based on the Chao1 richness estimator, the expected overall diversity of the sampled Peltigeralean mycobiont taxa and their cyanobionts in the studied montane forest ecosystem of Taita Hills would be approximately 90 species of mycobionts and over 200 cyanobiont variants (Table 1). According to this estimation, our sampling included approximately 83% of all mycobionts and 53% of all cyanobionts. The estimated coverage of *Nephroma* guild mycobionts was 96% and that of corresponding cyanobionts 73%, both values being higher than those estimated for the *Peltigera* guild, i.e., 82 and 47%, respectively.

**TABLE 1:** Diversity estimation.

	Observed		Chao1		
	N	S	S (est)	SD (est)	S/S (est)
Diversity of mycobionts	366	74	90	8.5	0.83
*Nephroma* guild mycobionts	162	22	23	1.6	0.96
*Peltigera* guild mycobionts	222	55	67	7.4	0.82
Diversity of cyanobionts	393	116	219	38.4	0.53
*Nephroma-*type *Nostoc* cyanobionts	156	22	30	8.2	0.73
*Collema/Peltigera-*type *Nostoc* cyanobionts	237	94	202	44.6	0.47

*N, number of specimens; S, number of mycobiont species or cyanobiont variants; S (est), Chao1 estimator of the expected number of species (Chao, 1984; Colwell et al., 2012); SD, standard deviation of S (est).*

Blast searches with the obtained trnL sequences revealed that three of the *Nephroma*-type *Nostoc* variants had been previously amplified from lichen-associated cyanobacteria: *Nostoc* variant N3 associating with several *Sticta* species in the Taita Hills has previously been sequenced from a *Crocodia* in Brazil and several *Nephroma* species in Japan (Stenroos et al., 2006; Fedrowitz et al., 2012, 2014); variant N7, in Taita Hills associating with *Crocodia, Sticta*, and *Leptogium* species, has previously been found from *Pseudocyphellaria* from Australia, Chile, and New Zealand (Summerfield et al., 2002; Summerfield and Eaton-Rye, 2006); and N18 found in Taita Hills from *Sticta* sp. 3 is previously reported from several *Nephroma* species from Japan (Fedrowitz et al., 2014). No sequences identical to any of the newly obtained *Collema*/*Peltigera*-type *Nostoc* trnL sequence were deposited in GenBank from other parts of the world.

### Lichen Guilds and Photobiont Sharing

**FIGURE 2:**
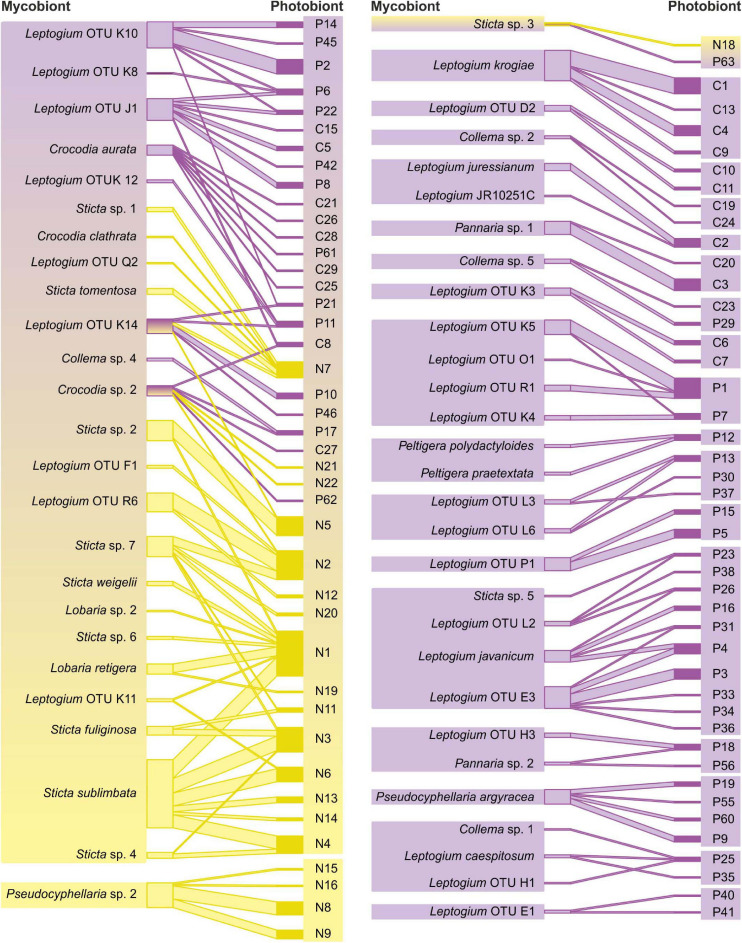
Mycobiont–photobiont associations among analyzed cyanolichens, excluding the 20 mycobiont–cyanobiont pairs that associated exclusively with one another. Mycobiont taxa on the left and cyanobiont variants on the right, yellow showing *Nephroma* guild lichens with *Nephroma*-type *Nostoc* variants, and purple indicating *Peltigera* guild lichens with *Collema*/*Peltigera*-type *Nostoc* variants. The line thickness between specific fungal species and cyanobiont variants is proportional to in how many different lichen specimens the interaction was detected.

Of all mycobiont species analyzed, 58% (43 species) shared at least one cyanobiont variant with some other mycobiont species (Figure 2). Cyanobionts were commonly shared between different fungal species, genera, and families (Figure 2 and Supplementary Figure 2). Even though the majority of species in most fungal genera participated in photobiont sharing, all *Pseudocyphellaria* species and the majority of *Pannaria* and *Collema* species each associated with their unique cyanobionts, and, in total, 20 mycobiont–cyanobiont pairs were exclusive with each other (Supplementary Figure 2 and Supplementary Table 2). When comparing the two photobiont-mediated guilds, the *Peltigera* guild included a larger number of cyanolichen specimens and mycobiont species and also cyanobiont variants (Supplementary Table 3). However, the most common epiphytic cyanolichen, *Sticta sublimbata* (Figures 1A,B), and several other abundant cyanolichens belonged to the *Nephroma* guild (Figure 2).

The regional *Nephroma* guild (i.e., mycobionts that associate with *Nephroma*-type *Nostoc*) in the Taita Hills included 22 species, mainly of the family Lobariaceae (*Lobaria*, *Pseudocyphellaria*, and *Sticta* spp.), but also five *Leptogium* species (Figure 2 and Supplementary Figure 2 and Supplementary Table 3). The regional *Peltigera* guild included 55 mycobiont species and was strongly dominated by *Leptogium* (>30 species), but also included species of, e.g., *Collema* and *Pannaria*. Not surprisingly, also all local *Peltigera* species associated with *Peltigera*-type cyanobionts and shared specific variants only between themselves. Nearly all cyanolichen mycobiont species were only members of one main guild (i.e., only associated with one main cyanobiont type). The only bipartite exception was the highly promiscuous, sexually reproducing *Leptogium* (OTU K14, morphotype cochleatum) and two cephalodiate lichens (*Crocodia* sp. 2 and *Sticta* sp. 3).

Generally, photobiont sharing was significantly (Fisher’s exact test *p* = 0.0071) more common among the *Nephroma* guild mycobionts (84% of species shared cyanobionts with at least one other species) than among the *Peltigera* guild mycobionts (48%) and also the species-specific interaction specialization (d’) among the *Nephroma* guild species (mean 0.52) was significantly (*t*-test *p* < 0.0001) lower than among the *Peltigera* guild mycobionts (mean 0.89). The structure of the bipartite interaction network, as well as several network structure qualifiers calculated separately for *Nephroma* guild and *Peltigera* guild taxa, revealed marked differences in mycobiont–photobiont interactions between the two guilds (Figure 2 and Table 2): In general, the interaction networks of both guilds had a nested structure: In *Nephroma* guild, the mycobiont interactions were highly significantly nested (*p* < 0.01, relative nestedness RN = 0.45), demonstrating that mycobionts associating with a restricted number of cyanobiont variants (specialists) tend to specifically associate with cyanobionts that are also housed by generalist mycobionts; however, the cyanobiont interactions within the *Nephroma* guild were not significantly nested (RN = 0.10). In the *Peltigera* guild, the mycobiont interactions were not significantly nested (RN = −0.11), but the cyanobiont interactions were (*p* < 0.05, RN = 0.33). Furthermore, the interaction network of the *Peltigera* guild was more compartmentalized (divided into more subunits not connected via mycobiont–photobiont interactions) than the *Nephroma* guild network. On the other hand, the relative numbers of mycobionts and cyanobionts were less skewed (web asymmetry) in the *Nephroma* guild than in the *Peltigera* guild, and also partner diversity and niche overlap of *Nephroma*-type *Nostoc* cyanobionts and niche overlap of *Nephroma* guild mycobionts were both markedly higher than in the interaction networks of the *Peltigera* guild.

**TABLE 2:** Network level information from the different lichen guilds, including the number of compartments (Com.) within the network not connected via mycobiont–photobiont interactions; connectance (Con.) indicating the proportion of realized interactions; NODF nestedness metric with *Z* score (*Z* > 1.64 indicates significance at *p* = 0.05), relative nestedness (RN) values, and whether the result differs significantly from a random matrix (Sig.), also separately to mycobionts (NODF my.) and cyanobionts (NODF cy.); specialization within the network (H_2_’), web asymmetry (Web asy.) indicating the difference between the numbers of mycobiont taxa and cyanobacterial variants; partner diversity (Partner div.) indicating the diversity and evenness of distribution among the interactions; and niche overlap indicating the similarity of interactions between taxa, i.e., the sharing of the mycobionts (My.) or cyanobionts (Cy.).

			NODF					Partner div.		Niche overlap	
	Com.	Con.	*Z*	RN	Sig.	H_2_′	Web asy.	My.	Cy.	My.	Cy.
*Nephroma* guild	5	0.089	1.80	0.27	Yes^a^	0.65	0.00	0.94	0.82	0.13	0.10
NODF my.			2.50	0.45	Yes^b^						
NODF cy.			0.51	0.10	No						
*Peltigera* guild	36	0.023	1.84	0.21	Yes^a^	0.81	0.21	0.82	0.24	0.01	0.02
NODF my.			−0.68	−0.11	No						
NODF cy.			2.19	0.33	Yes^a^						

*^*a*^*p* < 0.05; ^*b*^*p* < 0.01.*

### Fertile and Cephalodiate Lichens

The proportion of cyanobiont sharing taxa among the sexually reproducing (apotheciate) fungal species was slightly higher (78%) than in species with only symbiotic propagules (66%). Apotheciate taxa were also significantly (*t*-test *p* = 0.0453) less specialized in their cyanobiont interactions and associated with a higher diversity of cyanobiont variants than non-apotheciate and symbiotically reproducing taxa. Additionally, the abundantly fertile *Leptogium* OTU K14 (morphotype cochleatum) was the only bipartite cyanolichen species that associated with both *Nephroma* and *Peltigera*/*Collema*-type *Nostoc*. Also, the mycobionts of cephalodiate lichens were significantly (*t*-test *p* = 0.0005) less specialized in their cyanobiont interactions than those of bipartite lichens, with two out of four such mycobionts participating in both major guilds (Figure 2).

## Discussion

### Symbiotic Diversity and Guild Membership

The spectacular diversity of lichens has been highlighted in several recent studies from different parts of the Tropics (Lumbsch et al., 2011; Lücking et al., 2014; Moncada et al., 2014; Kaasalainen et al., 2021). Still, an overwhelming majority of tropical cyanolichens remains poorly known and probably even undescribed. This is especially true regarding the lichen cyanobionts, the diversity of which has hardly been studied in tropical regions. The montane forests of Taita Hills and Mt. Kasigau in SE Kenya together with other eastern African montane areas represent the global Eastern Afromontane biodiversity hotspot (Mittermeier et al., 2004).

The moist and relatively cool conditions of the montane forests also support high cyanolichen diversity, with a particularly high diversity of undescribed *Leptogium* species (Kaasalainen et al., 2021). Our results show that high genetic diversity is also observed in the cyanobacterial photobionts of the lichens, some of which are commonly shared between different fungal species, genera, and even families, giving rise to a complex network of interactions. Many of the cyanobacterial variants now detected have not been previously reported from other regions. Presumably, this is at least partially due to a general lack of sampling from the tropics and tropical montane environments, but it also suggests differentiation between regional cyanobiont pools of different climatic zones and/or geographic areas, this being in line with similar findings from other groups of micro-organisms (Martiny et al., 2006; Bahl et al., 2011; Tedersoo et al., 2014). This may indicate that regional lichen guilds tend to evolve around photobiont variants that are best adapted to local conditions (Rikkinen et al., 2002; Peksa and Skaloud, 2011; Piercey-Normore and Deduke, 2011; Onuţ-Brännström et al., 2018). The ability to select and switch between several symbionts can potentially promote ecological tolerance and evolutionary divergence (Muggia et al., 2014; Leavitt et al., 2015; Lutsak et al., 2016), and several studies have demonstrated examples of high reciprocal specificity between specific pairs of symbiont variants, often consistent over long geographical distances (Paulsrud et al., 2000; Otálora et al., 2010; Fedrowitz et al., 2011, 2012).

While most of the *Nostoc* variants identified from the Taita Hills are previously unknown, the main groups of *Nostoc* symbionts are very widespread also in temperate and boreal regions. *Nephroma*-type *Nostoc* has been found to associate with many species of *Lobaria*, *Nephroma*, *Pannaria*, *Pectenia*, *Pseudocyphellaria*, and *Sticta* in North and South America, Europe, Asia, and New Zealand (Lohtander et al., 2003; Summerfield and Eaton-Rye, 2006; Fedrowitz et al., 2011, 2012; Olsson et al., 2012; O’Brien et al., 2013). Also, *Collema*/*Peltigera*-type *Nostoc* variants have been identified from numerous cyanolichens in North and South America, Europe, Asia, and Antarctica (Rikkinen et al., 2002; Kaasalainen et al., 2012, 2015; O’Brien et al., 2013; Jüriado et al., 2019). The regional *Nephroma* guild in the Taita Hills is dominated by bipartite *Sticta* species and not by bipartite *Nephroma* species like in the forests of temperate and boreal Europe (Myllys et al., 2007; Fedrowitz et al., 2011, 2012). Similarly, while the *Peltigera* guild in boreal Europe and North America is typically dominated by various *Peltigera* species (Rikkinen et al., 2002; Myllys et al., 2007; Kaasalainen et al., 2012, 2015; O’Brien et al., 2013; Jüriado et al., 2019), the regional guild in the Taita Hills is strongly dominated by *Leptogium* species.

### Symbiotic Interaction Networks and Guild Ecology

On the level of individual mycobiont species, there was little overlap between the main guilds as most mycobionts were only associated with either *Nephroma* or *Collema*/*Peltigera* type cyanobionts. Conversely, many unrelated mycobionts from different genera shared cyanobionts. The structure of the interaction networks, e.g., nestedness and modularity, differed significantly between the two guilds, possibly reflecting ecological segregation between the different cyanobionts. The compartmentalization and lack of nestedness of networks are typical characteristics of intimate (high frequency of interactions between two species) and specialized symbiotic interaction networks (Blüthgen et al., 2007; Guimarães et al., 2007; Toju et al., 2015). The connected and nested interaction network structure is often observed in less intimate mutualistic interaction networks in which generalist species form the core of the guild and uncommon specialists interact mainly with dominant generalists (Bascompte et al., 2003). Generally, the nestedness of mutualistic networks tends to minimize competition, increase species diversity and abundances, and stabilize the structure of the interacting community (Bastolla et al., 2009; Suweis et al., 2013; Rohr et al., 2014).

**FIGURE 3:**
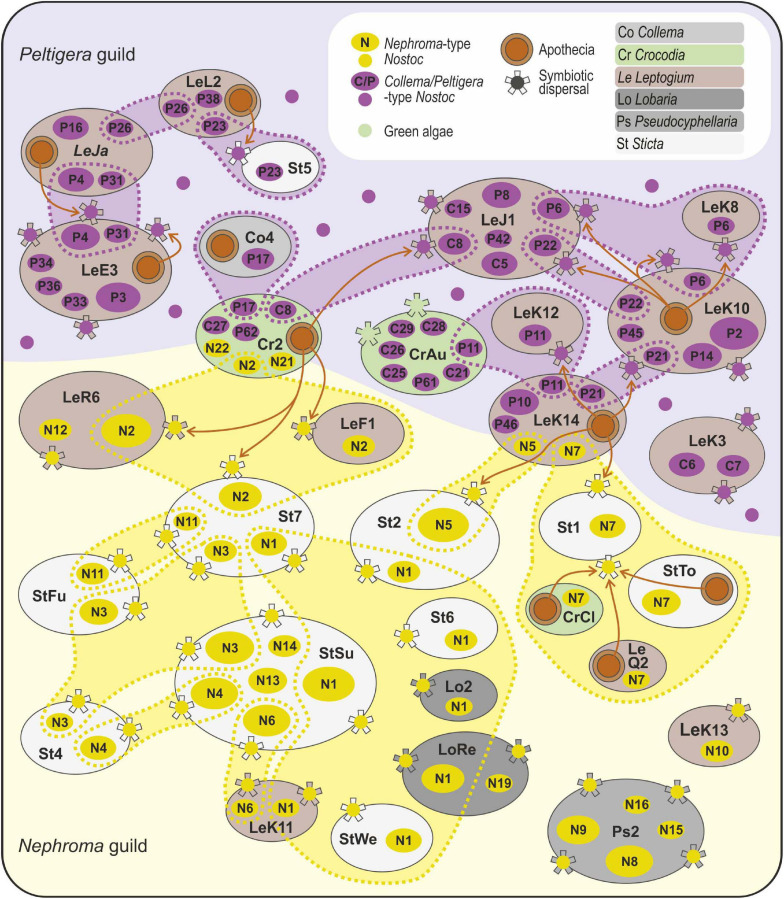
Schematic presentation of potential photobiont-mediated guild interactions among symbionts of Peltigeralean lichens in the Taita Hills, Kenya. The figure includes selected representatives of 74 different lichen species/OTUs identified in the study area. Each large oval represents a mycobiont taxon, the size proportional to the number of analyzed specimens and the color revealing generic affiliation (see legend); explanations for the species codes are available in Supplementary Table 2. The small ovals inside the mycobionts show the *Nostoc* variants identified from each mycobiont taxon (*Nephroma*-type cyanobionts in yellow, *Collema*/*Peltigera*-type cyanobionts in purple). The cyanobionts shared by several mycobiont species are enclosed with a dashed line. The observed reproductive mode of each lichen species is shown (apothecia as brown circles, symbiotic propagules as “stars”). The brown arrows indicate possible links between core species and fringe species.

Strong modularity and “anti-nestedness” now observed among the *Leptogium*-dominated mycobionts of *Peltigera* guild has previously been reported from an interaction network of *Peltigera* (Chagnon et al., 2018). This clearly suggests that the structural characters of the interactions may be a result of cyanobiont ecology rather than being determined by the mycobiont. A germinating fungal spore may obtain compatible photobionts from two different sources: either from other guild members or, if free-living cyanobionts exist, from the surrounding environment. Non-symbiotic *Nostoc* are common and sometimes abundant in many terrestrial environments (Dodds et al., 1995; Hrouzek et al., 2005), but the free-living variants generally differ from strains found in lichen thalli. Furthermore, studies on plant-symbiotic cyanobacteria have never reported finding *Nephroma*-type *Nostoc* variants from plant symbioses (Costa et al., 2004; Papaefthimiou et al., 2008; Rikkinen and Virtanen, 2008), and while they have been identified from literally hundreds of cyanolichen specimens from different ecosystems all around the world, not a single strain of this monophyletic group has so far been brought into axenic culture (Rikkinen, 2013). Hence, the *Nephroma*-type *Nostoc* variants may rarely if ever establish long-lasting free-living populations and appear to be primarily dispersed within the symbiotic propagules of lichens. On the other hand, some *Collema*/*Peltigera*-type *Nostoc* variants can be easily cultured and are closely related to plant symbionts and free-living *Nostoc* (Oksanen et al., 2002; Papaefthimiou et al., 2008; Rikkinen and Virtanen, 2008), and *Nostoc* strains similar to *Peltigera* cyanobionts have also been identified from lichen substrates (Zúñiga et al., 2017). Hence, the structural differences in the *Peltigera* and *Nephroma* guild interaction networks may be linked to differences in the general availability of compatible cyanobionts: possibly *Collema*/*Peltigera*-type *Nostoc* occur more commonly free-living in lichen substrates, while *Nephroma* guild mycobionts largely rely on lichen-mediated dispersal of their photobionts (Figure 3).

### Generalists and Specialists

Mutualistic communities typically include both generalists and specialists (Bascompte et al., 2003; Kostovcik et al., 2015). This pattern was also revealed here as the level of symbiont specificity varied from strictly reciprocally specific associations to varying degrees of promiscuity and symbiont sharing. Generally, apotheciate lichen species more commonly participated in photobiont sharing and exhibited lower photobiont specificity than symbiotically dispersing species. Also, several previous studies have reported lower levels of symbiont specificity among sexually reproducing lichens than in their symbiotically dispersing relatives (Myllys et al., 2007; Otálora et al., 2010; Fedrowitz et al., 2011; O’Brien et al., 2013).

The mycobionts of most cephalodiate lichens in the Taita Hills probably disperse without their cyanobacterial symbionts and tend to thus associate with a wider diversity of cyanobionts than bipartite lichens. Many of the *Nostoc* variants detected from cephalodiate lichens were found only once and rarely from bipartite cyanolichens, indicating that they more often represent “sporadic associates” and not well-established lichen symbionts. Previous studies have shown that while some cephalodiate lichens are relatively specific in their cyanobiont choice, others associate with a wide variety of *Nostoc* variants (Paulsrud et al., 2000; Lohtander et al., 2003; Myllys et al., 2007; Kaasalainen et al., 2009; O’Brien et al., 2013; La Pardo-De Hoz et al., 2018). On a general level, the comparatively low level of cyanobiont specificity of cephalodiate lichens may also relate to the different role of cyanobacteria in these lichens and bipartite cyanolichens. In the former, the dominant green algal photobionts are active in photosynthesis and the cyanobionts are responsible for nitrogen fixation (Hitch and Millbank, 1975).

### Facilitative Role of Photobiont-Mediated Guilds

Lichen guilds are hypothesized to include mycobiont species with different functional roles (Rikkinen, 2003). Typical core species [e.g., *Sticta sublimbata*, *Leptogium* OTUs J1 and K10 (morphotype cyanescens)] propagate and effectively distribute photobionts into the environment, thus facilitating the success of other guild members. The guilds may also include fringe species [e.g., *Leptogium javanicum* and *Leptogium* OTU K14 (morphotype cochleatum), *Crocodia* species] that largely depend on photobionts dispersed by core species or otherwise present in the environment (Figure 3). Symbiotically dispersing guild members have indeed been reported to facilitate the re-establishment of symbiosis in some sexually dispersing lichens (Belinchón et al., 2015; Svensson et al., 2016). As most symbiotically dispersing lichens are also capable of sexual reproduction, many lichen mycobionts likely function somewhere between the extremes; i.e., they both contribute to the common photobiont pool and periodically benefit from photobionts propagated and dispersed by other guild members. In some lichens, long-range dispersal is thought to mainly occur via fungal spores and short-range dispersal is thought to mainly occur via symbiotic propagules (Walser, 2004; Dal Grande et al., 2012), emphasizing the potential role of pre-existing guilds in facilitating long-range dispersal. The coexistence of several photobiont-mediated guilds may also increase the likelihood of occasional symbiont switches, which can significantly diversify the symbiotic community (Andrade-Domínguez et al., 2014).

## Data Availability Statement

The dataset generated and analyzed in this study can be found in NCBI GenBank (https://www.ncbi.nlm.nih.gov/nucleotide/). The accession numbers are listed in the Supplementary Material.

## Author Contributions

UK and JR designed the study, collected the specimens, and wrote the manuscript. GM, PP, and JR organized the fieldwork and research permit. UK and VT performed the laboratory work. UK and PP analyzed the data and prepared the figures and tables. All authors reviewed the manuscript.

## Conflict of Interest

The authors declare that the research was conducted in the absence of any commercial or financial relationships that could be construed as a potential conflict of interest.

## Supplementary Material

Supplementary Figure 1 Map of collection locations.

Supplementary Figure 2 Median-joining networks of the cyanobacterial trnL variants.

Supplementary Table 1 Lichen specimens.

Supplementary Table 2 Lichen species and their attributes.

Supplementary Table 3 Cyanobiont association patterns in the studied cyanolichen genera.

## References

[B1] Almeida-Neto M. Guimarães P. R. Loyola R. D. Ulrich W. (2008). A consistent metric for nestedness analysis in ecological systems: reconciling concept and measurement. *Oikos* 117 1227–1239. 10.1111/j.0030-1299.2008.16644.x

[B2] Altschul S. F. Gish W. Miller W. Myers E. W. Lipman D. J. (1990). Basic local alignment search tool. *J. Mol. Biol.* 215 403–410. 10.1016/S0022-2836(05)80360-2 2231712

[B3] Andrade-Domínguez A. Salazar E. Del Vargas-Lagunas M. C. Kolter R. Encarnación S. (2014). Eco-evolutionary feedbacks drive species interactions. *ISME J.* 8 1041–1054. 10.1038/ismej.2013.208 24304674 PMC3996687

[B4] Bahl J. Lau M. C. Y. Smith G. J. D. Vijaykrishna D. Cary S. C. Lacap D. C. (2011). Ancient origins determine global biogeography of hot and cold desert cyanobacteria. *Nat. Commun.* 2 163. 10.1038/ncom PMC3105302 21266963

[B5] Bandelt H. J. Forster P. Röhl A. (1999). Median-joining networks for inferring intraspecific phylogenies. *Mol. Biol. Evol.* 16 37–48. 10.1093/oxfordjournals.molbev.a026036 10331250

[B6] Bascompte J. Jordano P. Melián C. J. Olesen J. M. (2003). The nested assembly of plant-animal mutualistic networks. *Proc. Natl. Acad. Sci. U. S.A.* 100 9383–9387. 10.1073/pnas.1633576100 12881488 PMC170927

[B7] Bastolla U. Fortuna M. A. Pascual-García A. Ferrera A. Luque B. Bascompte J. (2009). The architecture of mutualistic networks minimizes competition and increases biodiversity. *Nature* 458 1018–1020. 10.1038/nature07950 19396144

[B8] Belinchón R. Yahr R. Ellis C. J. (2015). Interactions among species with contrasting dispersal modes explain distributions for epiphytic lichens. *Ecography* 38 762–768. 10.1111/ecog.01258

[B9] Blüthgen N. Menzel F. Hovestadt T. Fiala B. Blüthgen N. (2007). Specialization, constraints, and conflicting interests in mutualistic networks. *Curr. Biol.* 17 341–346. 10.1016/j.cub.2006.12.039 17275300

[B10] Bright M. Bulgheresi S. (2010). A complex journey: transmission of microbial symbionts. *Nat. Rev. Microbiol.* 8 218–230. 10.1038/nrmicro2262 20157340 PMC2967712

[B11] Chagnon P. L. Magain N. Miadlikowska J. Lutzoni F. (2018). Strong specificity and network modularity at a very fine phylogenetic scale in the lichen genus *Peltigera*. *Oecologia* 187 767–782. 10.1007/s00442-018-4159-6 29761320

[B12] Chao A. (1984). Nonparametric estimation of the number of classes in a population. *Scand. J. Statist.* 11 265–270.

[B13] Colwell R. K. (2013). *EstimateS: Statistical Estimation of Species Richness and Shared Species from Samples.* Available online at: http://purl.oclc.org/estimates

[B14] Colwell R. K. Chao A. Gotelli N. J. Lin S.-Y. Mao C. X. Chazdon R. L. (2012). Models and estimators linking individual-based and sample-based rarefaction, extrapolation and comparison of assemblages. *J. Plant Ecol.* 5 3–21. 10.1093/jpe/rtr044

[B15] Cornejo C. Scheidegger C. (2016). Cyanobacterial gardens: the liverwort Frullania asagrayana acts as a reservoir of lichen photobionts. *Environ. Microbiol. Rep.* 8 352–357. 10.1111/1758-2229.12386 26929112

[B16] Costa J.-L. Romero E. M. Lindblad P. (2004). Sequence based data supports a single *Nostoc strain* in individual coralloid roots of cycads. *FEMS Microbiol. Ecol.* 49 481–487. 10.1016/j.femsec.2004.05.001 19712296

[B17] Dahlkild Å Källersjö M. Lohtander K. Tehler A. (2001). Photobiont diversity in the physciaceae (Lecanorales). *Bryologist* 104 527–536. 10.1639/0007-2745(2001)104[0527:pditpl]2.0.co;2

[B18] Dal Forno M. Lawrey J. D. Sikaroodi M. Gillevet P. M. Schuettpelz E. Lücking R. (2020). Extensive photobiont sharing in a rapidly radiating cyanolichen clade. *Mol. Ecol.* 30 1755–1776. 10.1111/mec.15700 33080083

[B19] Dal Grande F. Beck A. Cornejo C. Singh G. Cheenacharoen S. Nelsen M. P. (2014). Molecular phylogeny and symbiotic selectivity of the green algal genus *Dictyochloropsis* s.l. (Trebouxiophyceae): a polyphyletic and widespread group forming photobiont-mediated guilds in the lichen family Lobariaceae. *New Phytol.* 202 455–470. 10.1111/nph.12678 24443895

[B20] Dal Grande F. Widmer I. Wagner H. H. Scheidegger C. (2012). Vertical and horizontal photobiont transmission within populations of a lichen symbiosis. *Mol. Ecol.* 21 3159–3172. 10.1111/j.1365-294X.2012.05482.x 22384938

[B21] Dodds W. K. Gudder D. A. Mollenhauer D. (1995). The ecology of nostoc. *J. Phycol.* 31 2–18. 10.1111/j.0022-3646.1995.00002.x

[B22] Dormann C. F. (2011). How to be a specialist? Quantifying specialisation in pollination networks. *Netw. Biol.* 1 1–20. 10.1163/9789004290228_002

[B23] Dormann C. F. Gruber B. Fründ J. (2008). Introducing the bipartite package analysing ecological networks. *R News* 8 8–11.

[B24] Dvořák P. Poulíèková A. Hašler P. Belli M. Casamatta D. A. Papini A. (2015). Species concepts and speciation factors in cyanobacteria, with connection to the problems of diversity and classification. *Biodivers. Conserv.* 24 739–757. 10.1007/s10531-015-0888-6

[B25] Enroth J. Nyqvist P. Malombe I. Pellikka P. K. Rikkinen J. (2013). Additions to the moss flora of the taita hills and mount kasigau, Kenya. *Pol. Bot. J.* 58 495–510. 10.2478/pbj-2013-0062

[B26] Enroth J. Pócs T. He X. Nyqvist P. Stam Å Malombe I. (2019). An annotated checklist of the bryophytes of Taita Hills region, Kenya. *Acta Mus. Sil. Sci. Nat.* 68 53–66. 10.2478/cszma-2019-0007

[B27] Ertz D. Guzow-Krzemińska B. Thor G. Łubek A. Kukwa M. (2018). Photobiont switching causes changes in the reproduction strategy and phenotypic dimorphism in the *Arthoniomycetes*. *Sci. Rep.* 8:4952. 10.1038/s41598-018-23219-3 29563606 PMC5862901

[B28] Fedrowitz K. Frisch A. Kaasalainen U. Ohmura Y. (2014). Nephroma squamigerum (Nephromataceae, Lichenized Ascomycota) Is a distinct species. *J. Jpn. Bot.* 89 346–354.

[B29] Fedrowitz K. Kaasalainen U. Rikkinen J. (2011). Genotype variability of *Nostoc symbionts* associated with three epiphytic Nephroma species in a boreal forest landscape. *Bryologist* 114 220–230. 10.1639/0007-2745-114.1.220

[B30] Fedrowitz K. Kaasalainen U. Rikkinen J. (2012). Geographic mosaic of symbiont selectivity in a genus of epiphytic cyanolichens. *Ecol. Evol.* 2 2291–2303. 10.1002/ece3.343 23139887 PMC3488679

[B31] Guimarães P. R. Rico-Gray V. Oliveira P. S. Izzo T. J. dos Reis S. F. Thompson J. N. (2007). Interaction intimacy affects structure and coevolutionary dynamics in mutualistic networks. *Curr. Biol.* 17 1797–1803. 10.1016/j.cub.2007.09.059 17949981

[B32] Hall T. A. (1999). BioEdit: a user-friendly biological sequence alignment editor and analysis program for Windows 95/98/NT. *Nucleic Acids Symp. Ser.* 41 95–98.

[B33] Hawksworth D. L. Grube M. (2020). Lichens redefined as complex ecosystems. *New Phytol.* 227 1281–1283. 10.1111/nph.16630 32484275 PMC7497170

[B34] Hitch C. J. B. Millbank J. W. (1975). Nitrogen metabolism in lichens. VII. Nitrogenase activity and heterocyst frequency in lichens with blue-green phycobionts. *New Phytol.* 75 239–244. 10.1111/j.1469-8137.1975.tb01392.x

[B35] Hrouzek P. Ventura S. Lukešová A. Mugnai M. A. Angela Turicchia S. Komárek J. (2005). Diversity of soil *Nostoc* strains: phylogenetic and phenotypic variability. *Arch. Hydrobiol. Suppl. Algol. Stud.* 117 251–264. 10.1127/1864-1318/2005/0117-0251

[B36] Jüriado I. Kaasalainen U. Jylhä M. Rikkinen J. (2019). Relationships between mycobiont identity, photobiont specificity and ecological preferences in the lichen genus *Peltigera* (Ascomycota) in Estonia (northeastern Europe). *Fungal Ecol.* 39 45–54. 10.1016/j.funeco.2018.11.005

[B37] Kaasalainen U. Fewer D. P. Jokela J. Wahlsten M. Sivonen K. Rikkinen J. (2012). Cyanobacteria produce a high variety of hepatotoxic peptides in lichen symbiosis. *Proc. Natl. Acad. Sci. U.S.A.* 109 5886–5891. 10.1073/pnas.1200279109 22451908 PMC3326460

[B38] Kaasalainen U. Jokela J. Fewer D. P. Sivonen K. Rikkinen J. (2009). Microcystin production in the tripartite cyanolichen *Peltigera leucophlebia*. *Mol. Plant Microbe Interact.* 22 695–702. 10.1094/MPMI-22-6-0695 19445594

[B39] Kaasalainen U. Olsson S. Rikkinen J. (2015). Evolution of the tRNALeu (UAA) Intron and congruence of genetic markers in lichen-symbiotic *Nostoc*. *PLoS One* 10:e0131223. 10.1371/journal.pone.0131223 26098760 PMC4476775

[B40] Kaasalainen U. Tuovinen V. Kirika P. M. Mollel N. P. Hemp A. Rikkinen J. (2021). Diversity of leptogium (Collemataceae, Ascomycota) in East African montane ecosystems. *Microorganisms* 9:314. 10.3390/microorganisms9020314 33546461 PMC7913733

[B41] Kopac S. Wang Z. Wiedenbeck J. Sherry J. Wu M. Cohan F. M. (2014). Genomic heterogeneity and ecological speciation within one subspecies of Bacillus subtilis. *Appl. Environ. Microbiol.* 80 4842–4853. 10.1128/AEM.00576-14 24907327 PMC4135754

[B42] Kostovcik M. Bateman C. C. Kolarik M. Stelinski L. L. Jordal B. H. Hulcr J. (2015). The ambrosia symbiosis is specific in some species and promiscuous in others: evidence from community pyrosequencing. *ISME J.* 9 126–138. 10.1038/ismej.2014.115 25083930 PMC4274425

[B43] La Pardo-De Hoz C. J. Magain N. Lutzoni F. Goward T. Restrepo S. Miadlikowska J. (2018). Contrasting symbiotic patterns in two closely related lineages of trimembered lichens of the genus *Peltigera*. *Front. Microbiol.* 9:2770. 10.3389/fmicb.2018.02770 30505297 PMC6250826

[B44] Leavitt S. D. Kraichak E. Nelsen M. P. Altermann S. Divakar P. K. Alors D. (2015). Fungal specificity and selectivity for algae play a major role in determining lichen partnerships across diverse ecogeographic regions in the lichen-forming family Parmeliaceae (Ascomycota). *Mol. Ecol.* 24 3779–3797. 10.1111/mec.13271 26073165

[B45] Lindgren H. Moncada B. Lücking R. Magain N. Simon A. Goffinet B. (2020). Cophylogenetic patterns in algal symbionts correlate with repeated symbiont switches during diversification and geographic expansion of lichen-forming fungi in the genus *Sticta* (Ascomycota, Peltigeraceae). *Mol. Phylogenet. Evol.* 150:106860. 10.1016/j.ympev.2020.106860 32473336

[B46] Lohtander K. Oksanen I. Rikkinen J. (2003). Genetic diversity of green algal and cyanobacterial photobionts in Nephroma (Peltigerales). *Lichenologist* 35 325–339. 10.1016/S0024-2829(03)00051-3

[B47] Lücking R. Dal-Forno M. Sikaroodi M. Gillevet P. M. Bungartz F. Moncada B. (2014). A single macrolichen constitutes hundreds of unrecognized species. *Proc. Natl. Acad. Sci. U.S.A.* 111 11091–11096. 10.1073/pnas.1403517111 24982168 PMC4121827

[B48] Lumbsch H. T. Ahti T. Altermann S. Paz G. A. de Aptroot A. Arup U. (2011). One hundred new species of lichenized fungi: a signature of undiscovered global diversity. *Phytotaxa* 18:1. 10.11646/phytotaxa.18.1.1

[B49] Lutsak T. Fernández-Mendoza F. Kirika P. M. Wondafrash M. Printzen C. (2016). Mycobiont-photobiont interactions of the lichen *Cetraria aculeata* in high alpine regions of East Africa and South America. *Symbiosis* 68 25–37. 10.1007/s13199-015-0351-1

[B50] Magain N. Miadlikowska J. Goffinet B. Sérusiaux E. Lutzoni F. (2017). Macroevolution of specificity in cyanolichens of the genus *Peltigera* section polydactylon (Lecanoromycetes, Ascomycota). *Syst. Biol.* 66 74–99. 10.1093/sysbio/syw065 28173598

[B51] Martiny J. B. H. Bohannan B. J. M. Brown J. H. Colwell R. K. Fuhrman J. A. Green J. L. (2006). Microbial biogeography: putting microorganisms on the map. *Nat. Rev. Microbiol.* 4 102–112. 10.1038/nrmicro1341 16415926

[B52] Mittermeier R. A. Robles G. P. Hoffmann M. Pilgrim J. Brooks T. Mittermeier C. G. (2004). *Hotspots revisited: Earth’s Biologically Richest and Most Endangered Ecoregiu9ons.* Mexico City: CEMEX.

[B53] Moncada B. Lücking R. Suárez A. (2014). Molecular phylogeny of the genus *Sticta* (lichenized Ascomycota: 85) in Colombia. *Fungal Divers.* 64 205–231. 10.1007/s13225-013-0230-0

[B54] Muggia L. Pérez-Ortega S. Kopun T. Zellnig G. Grube M. (2014). Photobiont selectivity leads to ecological tolerance and evolutionary divergence in a polymorphic complex of lichenized fungi. *Ann. Bot.* 114 463–475. 10.1093/aob/mcu146 25096324 PMC4204673

[B55] Müller K. F. Quandt D. Müller J. Neinhuis C. (2005). *PhyDe: Phylogenetic Data Editor.* Available online at: http://www.phyde.de/.

[B56] Myllys L. Stenroos S. Thell A. Kuusinen M. (2007). High cyanobiont selectivity of epiphytic lichens in old growth boreal forest of Finland. *New Phytol.* 173 621–629. 10.1111/j.1469-8137.2006.01944.x 17244057

[B57] NCBI Resource Coordinators (2016). Database resources of the national center for biotechnology information. *Nucleic Acids Res.* 44 D7–D19. 10.1093/nar/gkv1290 26615191 PMC4702911

[B58] Nyati S. Scherrer S. Werth S. Honegger R. (2014). Green-algal photobiont diversity (Trebouxia spp.) in representatives of Teloschistaceae (Lecanoromycetes, lichen-forming ascomycetes). *Lichenologist* 46 189–212. 10.1017/s0024282913000819

[B59] O’Brien H. E. Miadlikowska J. Lutzoni F. (2013). Assessing population structure and host specialization in lichenized cyanobacteria. *New Phytol.* 198 557–566. 10.1111/nph.12165 23406441

[B60] Oksanen I. Lohtander K. Paulsrud P. Rikkinen J. (2002). A molecular approach to cyanobacterial diversity in a rock-pool community involving gelatinous lichens and free-living Nostoc colonies. *Ann. Bot. Fenn.* 39 93–99.

[B61] Olsson S. Kaasalainen U. Rikkinen J. (2012). Reconstruction of structural evolution in the trnL intron P6b loop of symbiotic *Nostoc* (Cyanobacteria). *Curr. Genet.* 58 49–58. 10.1007/s00294-011-0364-0 22210193

[B62] Onuţ-Brännström I. Benjamin M. Scofield D. G. Heiðmarsson S. Andersson M. G. I Lindström E. S. (2018). Sharing of photobionts in sympatric populations of *Thamnolia* and *Cetraria lichens*: evidence from high-throughput sequencing. *Sci. Rep.* 8:4406. 10.1038/s41598-018-22470-y 29535321 PMC5849601

[B63] Otálora M. A. Martínez I. O’Brien H. E. Molina C. M. Aragón G. Lutzoni F. (2010). Multiple origins of high reciprocal symbiotic specificity at an intercontinental spatial scale among gelatinous lichens (Collemataceae, Lecanoromycetes). *Mol. Phylogenet. Evol.* 56 1089–1095. 10.1016/j.ympev.2010.05.013 20493269

[B64] Papaefthimiou D. Hrouzek P. Mugnai M. A. Lukešová A. Turicchia S. Rasmussen U. (2008). Differential patterns of evolution and distribution of the symbiotic behaviour in nostocacean cyanobacteria. *Int. J. Syst. Evol. Microbiol.* 58 553–564. 10.1099/ijs.0.65312-0 18319454

[B65] Paulsrud P. Lindblad P. (1998). Sequence variation of the tRNA(Leu) intron as a marker for genetic diversity and specificity of symbiotic cyanobacteria in some lichens. *Appl. Environ. Microbiol.* 64 310–315. 10.1128/AEM.64.1.310-315.1998 9435083 PMC124710

[B66] Paulsrud P. Rikkinen J. Lindblad P. (2000). Spatial patterns of photobiont diversity in some Nostoc-containing lichens. *New Phytol.* 146 291–299. 10.1046/j.1469-8137.2000.00647.x 33862966

[B67] Peksa O. Skaloud P. (2011). Do photobionts influence the ecology of lichens? A case study of environmental preferences in symbiotic green alga Asterochloris (Trebouxiophyceae). *Mol. Ecol.* 20 3936–3948. 10.1111/j.1365-294X.2011.05168.x 21699598

[B68] Piercey-Normore M. D. Deduke C. (2011). Fungal farmers or algal escorts: lichen adaptation from the algal perspective. *Mol. Ecol.* 20 3708–3710. 10.1111/j.1365-294X.2011.05191.x 21902745

[B69] R Development Core Team (2011). *R: A Language and Environment for Statistical Computing.* Vienna: R Foundation for Statistical Computing.

[B70] Rikkinen J. (2003). Ecological and evolutionary role of photobiont-mediated guilds in lichens. *Symbiosis* 34 99–110.

[B71] Rikkinen J. (2013). Molecular studies on cyanobacterial diversity in lichen symbioses. *MycoKeys* 6 3–32. 10.3897/mycokeys.6.3869

[B72] Rikkinen J. Oksanen I. Lohtander K. (2002). Lichen guilds share related cyanobacterial symbionts. *Science* 297 357. 10.1126/science.1072961 12130774

[B73] Rikkinen J. Virtanen V. (2008). Genetic diversity in cyanobacterial symbionts of thalloid bryophytes. *J. Exp. Bot.* 59 1013–1021. 10.1093/jxb/ern003 18325923

[B74] Rohr R. P. Saavedra S. Bascompte J. (2014). Ecological networks. on the structural stability of mutualistic systems. *Science* 345 1253497. 10.1126/science.1253497 25061214

[B75] Stam Å He X. Kaasalainen U. Toivonen M. Enroth J. Räsänen M. (2020). Epiphyte colonisation of fog nets in montane forests of the taita hills, Kenya. *Ann. Bot. Fenn* 57 227–238. 10.5735/085.057.0406

[B76] Stenroos S. Högnabba F. Myllys L. Hyvönen J. Thell A. (2006). High selectivity in symbiotic associations of lichenized ascomycetes and cyanobacteria. *Cladistics* 22 230–238. 10.1111/j.1096-0031.2006.00101.x

[B77] Strona G. Galli P. Seveso D. Montano S. Fattorini S. (2014). Nestedness for Dummies (n.d.): a user-friendly web interface for exploratory nestedness analysis. *J. Stat. Soft.* 59 1–9. 10.18637/jss.v059.c03

[B78] Suija A. Kaasalainen U. Kirika P. M. Rikkinen J. (2018). Taitaia, a novel lichenicolous fungus in tropical montane forests in Kenya (East Africa). *Lichenologist* 50 173–184. 10.1017/S0024282918000026

[B79] Summerfield T. C. Eaton-Rye J. J. (2006). *Pseudocyphellaria crocata*, P. neglecta and P. perpetua from the Northern and Southern Hemispheres are a phylogenetic species and share cyanobionts. *New Phytol.* 170 597–607. 10.1111/j.1469-8137.2006.01701.x 16626479

[B80] Summerfield T. C. Galloway D. J. Eaton-Rye J. J. (2002). Species of cyanolichens from *Pseudocyphellaria* with indistinguishable ITS sequences have different photobionts. *New Phytol.* 155 121–129. 10.1046/j.1469-8137.2002.00431.x 33873287

[B81] Suweis S. Simini F. Banavar J. R. Maritan A. (2013). Emergence of structural and dynamical properties of ecological mutualistic networks. *Nature* 500 449–452. 10.1038/nature12438 23969462

[B82] Svensson M. Caruso A. Yahr R. Ellis C. J. Thor G. Snäll T. (2016). Combined observational and experimental data provide limited support for facilitation in lichens. *Oikos* 125 278–283. 10.1111/oik.02279

[B83] Swinscow T. D. V. Krog H. (1988). *Macrolichens of East Africa.* London: Natural History Museum Publications.

[B84] Tedersoo L. Bahram M. Põlme S. Kõljalg U. Yorou N. S. Wijesundera R. (2014). Fungal biogeography. global diversity and geography of soil fungi. *Science* 346:1256688. 10.1126/science.1256688 25430773

[B85] Toju H. Guimarães P. R. Olesen J. M. Thompson J. N. (2015). Below-ground plant-fungus network topology is not congruent with above-ground plant-animal network topology. *Sci. Adv.* 1:e1500291. 10.1126/sciadv.1500291 26601279 PMC4646793

[B86] Walser J.-C. (2004). Molecular evidence for limited dispersal of vegetative propagules in the epiphytic lichen *Lobaria pulmonaria*. *Am. J. Bot.* 91 1273–1276. 10.3732/ajb.91.8.1273 21653485

[B87] Zúñiga C. Leiva D. Carú M. Orlando J. (2017). Substrates of peltigera lichens as a potential source of cyanobionts. *Microb. Ecol.* 74 561–569. 10.1007/s00248-017-0969-z 28349162

