# Supplementary material for: Complex Interaction Networks Among Cyanolichens of a Tropical Biodiversity Hotspot
Source: Front Microbiol. 2021 Jun 4;12:672333. doi: 10.3389/fmicb.2021.672333 (PMC8220813; doi:10.3389/fmicb.2021.672333)
Supplement: Supplementary Figure 1 — Map of collection locations. [file Data_Sheet_1.PDF]

# *Supplementary Material*

**Supplementary Table 1.** Lichen specimens with collection information and NCBI GenBank accession numbers for the trnL sequences. The naming of OTUs and clades in *Leptogium* follow Kaasalainen et al. (2021). All specimens were collected from Taita Hills or Mt, Kasigau, Kenya, in 2009−2011. In the trnL variant column, P = *Peltigera*-type *Nostoc*, C = *Collema*-type *Nostoc*, N = *Nephroma*-type *Nostoc*.

| Taxon | Collection number | Collection location | trnL variant | Accession number |
|---|---|---|---|---|
| *Collema* sp. 1 | JR10015 | Taita Research Station | P25 | KU317138 |
| *Collema* sp. 2 | JR10K246 | Mt. Kasigau | C24 | KU317139 |
| *Collema* sp. 2 | JR10272 | Macha | C30 | KU317140 |
| *Collema* sp. 3 | JR10064 | Shomoto Hill | C22 | KU317141 |
| *Collema* sp. 4 | JR10054C | Vuria | P17 | KU317142 |
| *Collema* sp. 4 | JR10115B | Vuria | P17 | KU317143 |
| *Collema* sp. 5 | JR10K073 | Mt. Kasigau | C23 | KU317144 |
| *Collema* sp. 5 | JR10K191 | Mt. Kasigau | P29 | KU317145 |
| *Collema* sp. 5 | JR10K022 | Mt. Kasigau | P29 | KU317146 |
| *Collema* sp. 6 | JR10K362 | Mt. Kasigau | P58 | KU317147 |
| *Collema* sp. 7 | JR10K093 | Mt. Kasigau | P57 | KU317148 |
| *Crocodia aurata* | JR10052A | Shomoto Hill | C21 | KU317149 |
| *Crocodia aurata* | JR10080C | Vuria | C25 | KU317150 |
| *Crocodia aurata* | UK110569b | Chawia | C26 | KU317151 |
| *Crocodia aurata* | JR_37B | Shomoto Hill | C28 | KU317152 |
| *Crocodia aurata* | JR_31B | Shomoto Hill | C29 | KU317153 |
| *Crocodia aurata* | UK110551 | Fururu | P11 | KU317154 |
| *Crocodia aurata* | JR09X6 | Chawia | P61 | KU317155 |
| *Crocodia clathrata* | JR_107 | Vuria | N7 | KU317163 |
| *Crocodia* sp. 2 | UK110539a | Vuria | C27 | KU317156 |
| *Crocodia* sp. 2 | JR110083 | Chawia | C8 | KU317157 |
| *Crocodia* sp. 2 | JR_150 | Iyale | N2 | KU317158 |
| *Crocodia* sp. 2 | JR_205A | Iyale | N21 | KU317159 |
| *Crocodia* sp. 2 | UK110569a | Chawia | N22 | KU317160 |
| *Crocodia* sp. 2 | JR110016B | Vuria | P17 | KU317161 |
| *Crocodia* sp. 2 | JR_78A | Vuria | P62 | KU317162 |
| *Leptogium caespitosum* | JR10016 | Taita Research Station | P25 | JX503550 |
| *Leptogium caespitosum* | JR10014A | Taita Research Station | P35 | JX503551 |
| *Leptogium javanicum* | JR10131B | Vuria | P16 | JX503665 |
| *Leptogium javanicum* | JR10131A | Vuria | P16 | JX503666 |
| *Leptogium javanicum* | JR10110A | Vuria | P26 | JX503668 |
| *Leptogium javanicum* | JR10115A | Vuria | P4 | JX503669 |
| *Leptogium javanicum* | JR10113A | Vuria | P31 | JX503670 |

| | | | | |
|---|---|---|---|---|
| *Leptogium javanicum* | JR10119B | Vuria | P4 | JX503671 |
| *Leptogium javanicum* | JR10113C | Vuria | P4 | JX503557 |
| *Leptogium javanicum* | JR10113B | Vuria | P16 | JX503667 |
| *Leptogium juressianum* | JR11067 | Fururu | C2 | JX503599 |
| *Leptogium juressianum* | JR11030C | Vuria | C2 | JX503600 |
| *Leptogium juressianum* | JR10072B | Vuria | C2 | JX503601 |
| *Leptogium juressianum* | JR11031A | Vuria | C2 | JX503602 |
| *Leptogium juressianum* | JR11050C | Vuria | C2 | JX503603 |
| *Leptogium krogiae* | JR10148 | Ngangao | C4 | JX503568 |
| *Leptogium krogiae* | JR10222A | Mwachora | C4 | JX503569 |
| *Leptogium krogiae* | JR_A45_R1 | Ngangao | C4 | JX503570 |
| *Leptogium krogiae* | JR10158B | Iyale | C4 | JX503571 |
| *Leptogium krogiae* | JR10192A | Iyale | C4 | JX503572 |
| *Leptogium krogiae* | JR10259A | Mwachora | C4 | JX503573 |
| *Leptogium krogiae* | JR10242 | Mwachora | C4 | JX503574 |
| *Leptogium krogiae* | JR10226A | Mwachora | C1 | JX503576 |
| *Leptogium krogiae* | JR10226B | Mwachora | C1 | JX503577 |
| *Leptogium krogiae* | JR10116A | Vuria | C1 | JX503581 |
| *Leptogium krogiae* | JR10252A | Mwachora | C13 | JX503582 |
| *Leptogium krogiae* | JR10088B | Vuria | C1 | JX503583 |
| *Leptogium krogiae* | JR10074A | Vuria | C9 | JX503584 |
| *Leptogium krogiae* | JR10182C | Iyale | C1 | JX503585 |
| *Leptogium krogiae* | JR10199A | Iyale | C9 | JX503586 |
| *Leptogium krogiae* | JR10201A | Iyale | C1 | JX503587 |
| *Leptogium krogiae* | JR10204 | Iyale | C1 | JX503588 |
| *Leptogium krogiae* | JR10210A | Iyale | C1 | JX503589 |
| *Leptogium krogiae* | JR11012B | Vuria | C1 | JX503590 |
| *Leptogium krogiae* | JR11039A | Vuria | C1 | JX503591 |
| *Leptogium krogiae* | JR11054A | Vuria | C1 | JX503592 |
| *Leptogium* OTU D2 | JR10072A | Vuria | C11 | JX503595 |
| *Leptogium* OTU D2 | JR10104 | Vuria | C10 | JX503596 |
| *Leptogium* OTU D2 | JR11030B | Vuria | C11 | JX503597 |
| *Leptogium* OTU D2 | JR11034B | Vuria | C10 | JX503598 |
| *Leptogium* OTU D3 | JR10252B | Mwachora | C14 | JX503594 |
| *Leptogium* OTU E1 | JR10061 | Shomoto Hill | P40 | JX503609 |
| *Leptogium* OTU E1 | JR10013B | Taita Research Station | P41 | JX503610 |
| *Leptogium* OTU E2 | JR10K012 | Mt. Kasigau | C12 | JX503606 |
| *Leptogium* OTU E2 | JR10020 | Taita Research Station | C12 | JX503607 |
| *Leptogium* OTU E3 | JR10126 | Vuria | P4 | JX503552 |
| *Leptogium* OTU E3 | JR10100 | Vuria | P4 | JX503553 |
| *Leptogium* OTU E3 | JR10120A | Vuria | P4 | JX503554 |
| *Leptogium* OTU E3 | JR10123A | Vuria | P36 | JX503555 |
| *Leptogium* OTU E3 | JR10124 | Vuria | P4 | JX503556 |
| *Leptogium* OTU E3 | JR10170B | Iyale | P3 | JX503539 |
| *Leptogium* OTU E3 | JR10283A | Macha | P3 | JX503540 |
| *Leptogium* OTU E3 | JR_X7B | Chawia | P33 | JX503541 |
| *Leptogium* OTU E3 | JR10156A | Iyale | P34 | JX503542 |

| | | | | |
|---|---|---|---|---|
| *Leptogium* OTU E3 | JR10156B | Iyale | P31 | JX503543 |
| *Leptogium* OTU E3 | JR10169A | Iyale | P3 | JX503544 |
| *Leptogium* OTU E3 | JR10260B | Mwachora | P3 | JX503545 |
| *Leptogium* OTU E3 | JR10059 | Shomoto Hill | P3 | JX503546 |
| *Leptogium* OTU E3 | JR10158A | Iyale | P3 | JX503547 |
| *Leptogium* OTU E3 | JR10170C | Iyale | P3 | JX503548 |
| *Leptogium* OTU F1 | JR10120C | Vuria | N2 | JX503633 |
| *Leptogium* OTU F1 | JR10170D | Iyale | N2 | JX503634 |
| *Leptogium* OTU H1 | JR10017a | Taita Research Station | P25 | JX503593 |
| *Leptogium* OTU H3 | JR10077 | Vuria | P18 | JX503630 |
| *Leptogium* OTU H3 | JR10163B | Iyale | P18 | JX503631 |
| *Leptogium* OTU H3 | JR10081A | Vuria | P18 | JX503632 |
| *Leptogium* OTU J1 | JR11010Aa | Vuria | P6 | JX503611 |
| *Leptogium* OTU J1 | JR10097B | Vuria | P6 | JX503612 |
| *Leptogium* OTU J1 | JR_X1 | Chawia | C8 | JX503613 |
| *Leptogium* OTU J1 | JR_D12_B1 | Ngangao | C8 | JX503614 |
| *Leptogium* OTU J1 | JR10186A | Iyale | P22 | JX503615 |
| *Leptogium* OTU J1 | JR10128B | Vuria | P8 | JX503616 |
| *Leptogium* OTU J1 | JR10186C | Iyale | P22 | JX503617 |
| *Leptogium* OTU J1 | UK110521 | Vuria | P8 | JX503618 |
| *Leptogium* OTU J1 | JR11009A | Vuria | P8 | JX503619 |
| *Leptogium* OTU J1 | JR10232B | Mwachora | P8 | JX503620 |
| *Leptogium* OTU J1 | JR_Y9 | Mbololo | P42 | JX503621 |
| *Leptogium* OTU J1 | JR10146 | Ngangao | C5 | JX503622 |
| *Leptogium* OTU J1 | JR11006A | Vuria | C5 | JX503623 |
| *Leptogium* OTU J1 | JR_AT1 | Ngangao | C15 | JX503626 |
| *Leptogium* OTU J1 | JR_AT2A | Ngangao | C5 | JX503629 |
| *Leptogium* OTU K1 | JR_W8a | Mt. Kasigau | P43 | JX503637 |
| *Leptogium* OTU K3 | JR10098A | Vuria | C6 | JX503638 |
| *Leptogium* OTU K3 | JR10098B | Vuria | C6 | JX503639 |
| *Leptogium* OTU K3 | JR10096 | Vuria | C7 | JX503641 |
| *Leptogium* OTU K3 | JR_Y15 | Ngangao | C7 | JX503642 |
| *Leptogium* OTU K3 | JR10094 | Vuria | C6 | JX503640 |
| *Leptogium* OTU K4 | JR10158C | Iyale | P7 | JX503645 |
| *Leptogium* OTU K4 | JR10177C | Iyale | P7 | JX503646 |
| *Leptogium* OTU K4 | JR10259B | Mwachora | P7 | JX503647 |
| *Leptogium* OTU K5 | JR11060 | Fururu | P7 | JX503649 |
| *Leptogium* OTU K5 | JR10239A | Mwachora | P1 | JX503650 |
| *Leptogium* OTU K5 | JR11054D | Vuria | P1 | JX503653 |
| *Leptogium* OTU K5 | JR10030A | Shomoto Hill | P1 | JX503651 |
| *Leptogium* OTU K5 | JR10033A | Shomoto Hill | P1 | JX503652 |
| *Leptogium* OTU K5 | JR10251A | Mwachora | P1 | JX503654 |
| *Leptogium* OTU K5 | JR11002A | Werugha | P1 | JX503655 |
| *Leptogium* OTU K5 | JR11026 | Vuria | P1 | JX503656 |
| *Leptogium* OTU K5 | UK110513 | Vuria | P1 | JX503657 |
| *Leptogium* OTU K5 | JR10016F | Vuria | P1 | JX503658 |
| *Leptogium* OTU K6 | JR_X7A | Chawia | P44 | JX503648 |
| *Leptogium* OTU K7 | JR10125 | Vuria | P32 | JX503663 |

| | | | | |
|---|---|---|---|---|
| *Leptogium* OTU K8 | JR10145A | Ngangao | P6 | JX503660 |
| *Leptogium* OTU K10 | JR10169C | Iyale | P2 | JX503672 |
| *Leptogium* OTU K10 | JR10022C | Taita Research Station | P45 | JX503675 |
| *Leptogium* OTU K10 | JR10197 | Iyale | P2 | JX503676 |
| *Leptogium* OTU K10 | JR10110C | Vuria | P2 | JX503677 |
| *Leptogium* OTU K10 | JR10128A | Vuria | P6 | JX503678 |
| *Leptogium* OTU K10 | JR10178B | Iyale | P2 | JX503679 |
| *Leptogium* OTU K10 | JR10221A | Mwachora | P14 | JX503680 |
| *Leptogium* OTU K10 | JR10221B | Mwachora | P14 | JX503681 |
| *Leptogium* OTU K10 | JR11078A | Chawia | P2 | JX503683 |
| *Leptogium* OTU K10 | JR10199B | Iyale | P2 | JX503684 |
| *Leptogium* OTU K10 | JR10283C | Macha | P14 | JX503685 |
| *Leptogium* OTU K10 | JR10269A | Mwachora | P22 | JX503686 |
| *Leptogium* OTU K10 | JR10269B | Mwachora | P21 | JX503687 |
| *Leptogium* OTU K10 | JR10149B | Iyale | P2 | JX503689 |
| *Leptogium* OTU K10 | JR10153A | Iyale | P2 | JX503690 |
| *Leptogium* OTU K10 | JR10185A | Iyale | P2 | JX503691 |
| *Leptogium* OTU K10 | JR10174A | Iyale | P2 | JX503674 |
| *Leptogium* OTU K10 | JR10235A | Mwachora | P14 | JX503682 |
| *Leptogium* OTU K11 | JR10040B | Shomoto Hill | N1 | JX503692 |
| *Leptogium* OTU K11 | JR10239C | Mwachora | N6 | JX503693 |
| *Leptogium* OTU K12 | UK110511 | Vuria | P11 | JX503698 |
| *Leptogium* OTU K12 | JR_Ar1 | Ngangao | P11 | JX503699 |
| *Leptogium* OTU K13 | JR10218A | Iyale | N10 | JX503695 |
| *Leptogium* OTU K13 | JR10218B | Iyale | N10 | JX503696 |
| *Leptogium* OTU K14 | JR10134A | Ngangao | P10 | JX503701 |
| *Leptogium* OTU K14 | JR10114A | Vuria | N7 | JX503702 |
| *Leptogium* OTU K14 | JR10210B | Iyale | P10 | JX503704 |
| *Leptogium* OTU K14 | JR_Z7B | Ngangao | P46 | JX503705 |
| *Leptogium* OTU K14 | JR_D12_B2 | Ngangao | N5 | JX503706 |
| *Leptogium* OTU K14 | UK110507 | Ngangao | P11 | JX503707 |
| *Leptogium* OTU K14 | JR10136A | Ngangao | P10 | JX503708 |
| *Leptogium* OTU K14 | UK110505B | Ngangao | P21 | JX503709 |
| *Leptogium* OTU K14 | JR10136B | Ngangao | P10 | JX503710 |
| *Leptogium* OTU L2 | JR10099 | Vuria | P26 | JX503565 |
| *Leptogium* OTU L2 | JR10161 | Iyale | P23 | JX503566 |
| *Leptogium* OTU L2 | JR11087 | Chawia | P38 | JX503567 |
| *Leptogium* OTU L3 | JR11094B | Chawia | P37 | JX503558 |
| *Leptogium* OTU L3 | JR10182A | Iyale | P13 | JX503559 |
| *Leptogium* OTU L3 | JR10152A | Iyale | P13 | JX503560 |
| *Leptogium* OTU L6 | JR10279A | Macha | P13 | JX503561 |
| *Leptogium* OTU L6 | JR10178A | Iyale | P30 | JX503562 |
| *Leptogium* OTU L6 | JR10149A | Iyale | P13 | JX503564 |
| *Leptogium* OTU O1 | JR11004B | Werugha | P1 | JX503743 |
| *Leptogium* OTU P1 | JR10021 | Taita Research Station | P15 | JX503712 |
| *Leptogium* OTU P1 | JR10054 | Shomoto Hill | P15 | JX503713 |
| *Leptogium* OTU P1 | JR10062 | Shomoto Hill | P15 | JX503714 |

| | | | | |
|---|---|---|---|---|
| *Leptogium* OTU P1 | JR10012 | Taita Research Station | P5 | JX503711 |
| *Leptogium* OTU P1 | JR10222B | Mwachora | P5 | JX503715 |
| *Leptogium* OTU P1 | JR10036 | Shomoto Hill | P5 | JX503716 |
| *Leptogium* OTU P1 | JR10063A | Shomoto Hill | P5 | JX503717 |
| *Leptogium* OTU P1 | JR10022B | Taita Research Station | P5 | JX503718 |
| *Leptogium* OTU P1 | JR10022A | Taita Research Station | P5 | JX503719 |
| *Leptogium* OTU Q1 | JR10019A | Taita Research Station | P49 | JX503746 |
| *Leptogium* OTU Q2 | JR10088A | Vuria | N7 | JX503703 |
| *Leptogium* OTU R1 | JR10030B | Shomoto Hill | P1 | JX503739 |
| *Leptogium* OTU R1 | JR11001 | Werugha | P1 | JX503740 |
| *Leptogium* OTU R1 | JR10277B | Macha | P1 | JX503741 |
| *Leptogium* OTU R1 | JR11004A | Werugha | P1 | JX503742 |
| *Leptogium* OTU R2 | JR10038B | Shomoto Hill | C18 | JX503737 |
| *Leptogium* OTU R3 | JR10186B | Iyale | C17 | JX503736 |
| *Leptogium* OTU R6 | JR11010B | Vuria | N12 | JX503721 |
| *Leptogium* OTU R6 | JR10206A | Iyale | N2 | JX503722 |
| *Leptogium* OTU R6 | JR10206B | Iyale | N2 | JX503723 |
| *Leptogium* OTU R6 | JR10260A | Mwachora | N2 | JX503724 |
| *Leptogium* OTU R6 | JR10279C | Macha | N2 | JX503725 |
| *Leptogium* OTU R6 | JR11009B | Vuria | N2 | JX503726 |
| *Leptogium* OTU R6 | JR10040A | Shomoto Hill | N2 | JX503727 |
| *Leptogium* OTU R6 | JR10050 | Shomoto Hill | N2 | JX503728 |
| *Leptogium* OTU R6 | JR10053C | Shomoto Hill | N2 | JX503729 |
| *Leptogium* OTU R6 | JR10041Aa | Shomoto Hill | N2 | JX503730 |
| *Leptogium* OTU R6 | JR11088B | Chawia | N12 | JX503731 |
| *Leptogium* OTU R6 | JR10035 | Shomoto Hill | N2 | JX503732 |
| *Leptogium* OTU R6 | JR10033B | Shomoto Hill | N2 | JX503733 |
| *Leptogium* OTU R7 | JR_W9 | Mt. Kasigau | P28 | JX503734 |
| *Leptogium* OTU R7 | JR_W5B | Mt. Kasigau | P28 | JX503735 |
| *Leptogium* sp. (Clade E) | JR11094A | Chawia | P39 | JX503608 |
| *Leptogium* sp. (Clade K) | JR10251C | Mwachora | C2 | JX503644 |
| *Leptogium* sp. (Clade R) | JR_W1B_R2 | Mt. Kasigau | C16 | JX503720 |
| *Leptogium* sp. | JR_X9 | Chawia | P3 | JX503549 |
| *Leptogium* sp. | JR10119A | Vuria | P30 | JX503563 |
| *Leptogium* sp. | JR10255 | Mwachora | C4 | JX503575 |
| *Leptogium* sp. | JR10226C | Mwachora | C1 | JX503578 |
| *Leptogium* sp. | JR_CA1 | Ngangao | C1 | JX503579 |
| *Leptogium* sp. | JR_D8B | Ngangao | C1 | JX503580 |
| *Leptogium* sp. | JR10252C | Mwachora | C2 | JX503604 |
| *Leptogium* sp. | JR_A57 | Ngangao | C5 | JX503624 |
| *Leptogium* sp. | JR10038A | Shomoto Hill | N2 | JX503635 |
| *Leptogium* sp. | JR10165 | Iyale | N2 | JX503636 |
| *Leptogium* sp. | JR_Z7A | Ngangao | C7 | JX503643 |
| *Leptogium* sp. | JR10145B | Ngangao | P6 | JX503661 |
| *Leptogium* sp. | JR10088C | Vuria | P2 | JX503673 |
| *Leptogium* sp. | JR_Z10 | Ngangao | P21 | JX503688 |
| *Leptogium* sp. | JR_W5C_R1 | Mt. Kasigau | N6 | JX503694 |
| *Leptogium* sp. | JR10218C | Iyale | N10 | JX503697 |

| | | | | |
|---|---|---|---|---|
| *Leptogium* sp. | JR10239B | Mwachora | P47 | JX503744 |
| *Leptogium* sp. | JR10028 | Shomoto Hill | P48 | JX503745 |
| *Leptogium* sp. | JR10014B | Taita Research Station | P50 | JX503747 |
| *Leptogium* sp. | JR10251B | Mwachora | P51 | JX503748 |
| *Leptogium* sp. | JR_W1 | Mt. Kasigau | P52 | JX503749 |
| *Leptogium* sp. | JR_Z16 | Ngangao | P53 | JX503750 |
| *Leptogium* sp. | JR_W2B | Mt. Kasigau | P54 | JX503751 |
| *Leptogium* sp. | JR11094D | Chawia | P7 | JX503756 |
| *Leptogium* sp. | JR10248A | Mwachora | C19 | JX503757 |
| *Lobaria retigera* | JR10140B | Ngangao | N1 | KU317164 |
| *Lobaria retigera* | JR10140C | Ngangao | N1 | KU317165 |
| *Lobaria retigera* | JR110058D | Vuria | N1 | KU317166 |
| *Lobaria retigera* | JR10075A | Vuria | N1 | KU317167 |
| *Lobaria retigera* | JR10075C | Vuria | N1 | KU317168 |
| *Lobaria retigera* | JR10140A | Ngangao | N1 | KU317169 |
| *Lobaria retigera* | JR10102 | Vuria | N19 | KU317170 |
| *Lobaria* sp. 2 | JR_Z13 | Ngangao | N1 | KU317171 |
| *Pannaria* sp. 1 | JR110093A | Chawia | C20 | KU317172 |
| *Pannaria* sp. 1 | JR09A51 | Ngangao | C3 | KU317173 |
| *Pannaria* sp. 1 | JR09A54 | Ngangao | C3 | KU317174 |
| *Pannaria* sp. 1 | JR09A56 | Ngangao | C3 | KU317175 |
| *Pannaria* sp. 1 | JR09T2b | Ngangao | C3 | KU317176 |
| *Pannaria* sp. 1 | JR09Z8 | Ngangao | C3 | KU317177 |
| *Pannaria* sp. 1 | JR09Z11A | Ngangao | C3 | KU317178 |
| *Pannaria* sp. 1 | JR09Y2b | Mbololo | C3 | KU317179 |
| *Pannaria* sp. 1 | JR110008 | Vuria | C3 | KU317180 |
| *Pannaria* sp. 2 | JR10058A | Vuria | P18 | KU317181 |
| *Pannaria* sp. 2 | UK110524a | Vuria | P56 | KU317182 |
| *Pannaria* sp. 3 | JR110033 | Vuria | P27 | KU317183 |
| *Pannaria* sp. 3 | UK110524e | Vuria | P27 | KU317184 |
| *Pannaria* sp. 4 | JR10K256A | Mt. Kasigau | P20 | KU317185 |
| *Pannaria* sp. 4 | JR10K256B | Mt. Kasigau | P20 | KU317186 |
| *Pannaria* sp. 4 | JR10K256C | Mt. Kasigau | P20 | KU317187 |
| *Pannaria* sp. 5 | UK110562 | Chawia | P59 | KU317188 |
| *Peltigera dolichorhiza* | JR10085A | Vuria | P24 | KU317189 |
| *Peltigera dolichorhiza* | UK110536 | Vuria | P24 | KU317190 |
| *Peltigera polydactyloides* | UK110525 | Vuria | P12 | KU317191 |
| *Peltigera polydactyloides* | UK110526 | Vuria | P12 | KU317192 |
| *Peltigera praetextata* | UK110518 | Vuria | P12 | KU317193 |
| *Peltigera praetextata* | JR110035 | Vuria | P12 | KU317194 |
| *Pseudocyphellaria argyraceae* | JR09A35 | Ngangao | P19 | KU317195 |
| *Pseudocyphellaria argyraceae* | JR09Y2a | Mbololo | P19 | KU317196 |
| *Pseudocyphellaria argyraceae* | JR09Z6 | Ngangao | P19 | KU317197 |
| *Pseudocyphellaria argyraceae* | JR09Y6 | Mbololo | P55 | KU317198 |
| *Pseudocyphellaria argyraceae* | UK110538a | Vuria | P60 | KU317199 |
| *Pseudocyphellaria argyraceae* | JR_K257 | Mt. Kasigau | P60 | KU317200 |
| *Pseudocyphellaria argyraceae* | JR09A59 | Ngangao | P9 | KU317201 |

| | | | | |
|---|---|---|---|---|
| *Pseudocyphellaria argyraceae* | JR09D13A | Ngangao | P9 | KU317202 |
| *Pseudocyphellaria argyraceae* | JR09X2 | Chawia | P9 | KU317203 |
| *Pseudocyphellaria argyraceae* | JR09X5 | Chawia | P9 | KU317204 |
| *Pseudocyphellaria* sp. 2 | JR10237B | Mwachora | N15 | KU317205 |
| *Pseudocyphellaria* sp. 2 | JR10228C | Mwachora | N16 | KU317206 |
| *Pseudocyphellaria* sp. 2 | JR09X10 | Chawia | N8 | KU317207 |
| *Pseudocyphellaria* sp. 2 | JR09W1Ba | Mt. Kasigau | N8 | KU317208 |
| *Pseudocyphellaria* sp. 2 | JR10233A | Mwachora | N8 | KU317209 |
| *Pseudocyphellaria* sp. 2 | JR10237C | Mwachora | N8 | KU317210 |
| *Pseudocyphellaria* sp. 2 | JR10261B | Mwachora | N8 | KU317211 |
| *Pseudocyphellaria* sp. 2 | JR10223B | Mwachora | N8 | KU317212 |
| *Pseudocyphellaria* sp. 2 | JR10233B | Mwachora | N8 | KU317213 |
| *Pseudocyphellaria* sp. 2 | JR10237A | Mwachora | N8 | KU317214 |
| *Pseudocyphellaria* sp. 2 | JR10065B | Shomoto Hill | N8 | KU317215 |
| *Pseudocyphellaria* sp. 2 | JR10261A | Mwachora | N9 | KU317216 |
| *Pseudocyphellaria* sp. 2 | JR10228B | Mwachora | N9 | KU317217 |
| *Pseudocyphellaria* sp. 2 | JR10154A | Iyale | N9 | KU317218 |
| *Pseudocyphellaria* sp. 2 | JR10154B | Iyale | N9 | KU317219 |
| *Pseudocyphellaria* sp. 2 | JR10154C | Iyale | N9 | KU317220 |
| *Pseudocyphellaria* sp. 2 | UK110504a | Ngangao | N9 | KU317221 |
| *Pseudocyphellaria* sp. 3 | JR10190A | Iyale | N17 | KU317222 |
| *Sticta fuliginosa* | JR09A46 | Ngangao | N11 | KU317264 |
| *Sticta fuliginosa* | JR10076 | Vuria | N11 | KU317265 |
| *Sticta fuliginosa* | JR10245 | Mwachora | N3 | KU317266 |
| *Sticta fuliginosa* | JR09D21B | Ngangao | N3 | KU317267 |
| *Sticta fuliginosa* | JR10190B | Iyale | N3 | KU317268 |
| *Sticta fuliginosa* | JR10151 | Iyale | N3 | KU317269 |
| *Sticta sublimbata* | JR09Y15b | Mbololo | N1 | KU317270 |
| *Sticta sublimbata* | JR09Y12a | Mbololo | N1 | KU317271 |
| *Sticta sublimbata* | JR10253B | Mwachora | N1 | KU317272 |
| *Sticta sublimbata* | JR10253C | Mwachora | N1 | KU317273 |
| *Sticta sublimbata* | JR10253A | Mwachora | N1 | KU317274 |
| *Sticta sublimbata* | JR10147B | Ngangao | N1 | KU317275 |
| *Sticta sublimbata* | JR10147A | Ngangao | N1 | KU317276 |
| *Sticta sublimbata* | JR09D6A | Ngangao | N1 | KU317277 |
| *Sticta sublimbata* | JR09Z2 | Ngangao | N1 | KU317278 |
| *Sticta sublimbata* | JR10195 | Iyale | N1 | KU317279 |
| *Sticta sublimbata* | JR10208A | Iyale | N1 | KU317280 |
| *Sticta sublimbata* | JR10214B | Iyale | N1 | KU317281 |
| *Sticta sublimbata* | JR10216C | Iyale | N1 | KU317282 |
| *Sticta sublimbata* | JR09W5Cb | Mt. Kasigau | N13 | KU317283 |
| *Sticta sublimbata* | JR09W12 | Mt. Kasigau | N13 | KU317284 |
| *Sticta sublimbata* | UK110547a | Fururu | N13 | KU317285 |
| *Sticta sublimbata* | UK110547b | Fururu | N13 | KU317286 |
| *Sticta sublimbata* | JR10281C | Macha | N14 | KU317287 |
| *Sticta sublimbata* | JR10282B | Macha | N14 | KU317288 |
| *Sticta sublimbata* | JR10276N | Macha | N3 | KU317289 |
| *Sticta sublimbata* | JR10220B | Mwachora | N3 | KU317290 |

| | | | | |
|---|---|---|---|---|
| *Sticta sublimbata* | JR10220D | Mwachora | N3 | KU317291 |
| *Sticta sublimbata* | JR09Z4 | Ngangao | N3 | KU317292 |
| *Sticta sublimbata* | JR09A60 | Ngangao | N3 | KU317293 |
| *Sticta sublimbata* | JR10066C | Shomoto Hill | N3 | KU317294 |
| *Sticta sublimbata* | JR10039A | Shomoto Hill | N3 | KU317295 |
| *Sticta sublimbata* | JR10039B | Shomoto Hill | N3 | KU317296 |
| *Sticta sublimbata* | JR10039C | Shomoto Hill | N3 | KU317297 |
| *Sticta sublimbata* | JR10066A | Shomoto Hill | N3 | KU317298 |
| *Sticta sublimbata* | JR10228D | Mwachora | N4 | KU317299 |
| *Sticta sublimbata* | JR10230A | Mwachora | N4 | KU317300 |
| *Sticta sublimbata* | JR10230B | Mwachora | N4 | KU317301 |
| *Sticta sublimbata* | JR09Z1 | Ngangao | N4 | KU317302 |
| *Sticta sublimbata* | JR10184 | Iyale | N4 | KU317303 |
| *Sticta sublimbata* | JR10191 | Iyale | N4 | KU317304 |
| *Sticta sublimbata* | JR10216A | Iyale | N4 | KU317305 |
| *Sticta sublimbata* | JR10216B | Iyale | N4 | KU317306 |
| *Sticta sublimbata* | JR09D5 | Ngangao | N4 | KU317307 |
| *Sticta sublimbata* | JR09W11 | Mt. Kasigau | N6 | KU317308 |
| *Sticta sublimbata* | JR10220A | Mwachora | N6 | KU317309 |
| *Sticta sublimbata* | JR10234A | Mwachora | N6 | KU317310 |
| *Sticta sublimbata* | JR10241 | Mwachora | N6 | KU317311 |
| *Sticta sublimbata* | JR10262A | Mwachora | N6 | KU317312 |
| *Sticta sublimbata* | JR10262B | Mwachora | N6 | KU317313 |
| *Sticta sublimbata* | JR10066B | Shomoto Hill | N6 | KU317314 |
| *Sticta sublimbata* | JR10032A | Shomoto Hill | N6 | KU317315 |
| *Sticta sublimbata* | JR10032B | Shomoto Hill | N6 | KU317316 |
| *Sticta tomentosa* | JR10095A | Vuria | N7 | KU317321 |
| *Sticta tomentosa* | JR10122 | Vuria | N7 | KU317322 |
| *Sticta tomentosa* | UK110523b | Vuria | N7 | KU317323 |
| *Sticta tomentosa* | JR10086 | Vuria | N7 | KU317324 |
| *Sticta weigelii* | JR090X3 | Chawia | N1 | KU317317 |
| *Sticta weigelii* | JR10212B | Iyale | N1 | KU317318 |
| *Sticta weigelii* | JR10180B | Iyale | N1 | KU317319 |
| *Sticta* sp. 1 | JR10112 | Vuria | N7 | KU317225 |
| *Sticta* sp. 1 | JR10121B | Vuria | N7 | KU317226 |
| *Sticta* sp. 1 | JR10189 | Iyale | N7 | KU317227 |
| *Sticta* sp. 2 | JR10193A | Iyale | N1 | KU317244 |
| *Sticta* sp. 2 | JR10193B | Iyale | N1 | KU317245 |
| *Sticta* sp. 2 | JR_58:7 | Vuria | N5 | KU317246 |
| *Sticta* sp. 2 | JR09D12A | Ngangao | N5 | KU317247 |
| *Sticta* sp. 2 | JR110055 | Vuria | N5 | KU317248 |
| *Sticta* sp. 2 | JR10028B | Vuria | N5 | KU317249 |
| *Sticta* sp. 2 | JR110030A | Vuria | N5 | KU317250 |
| *Sticta* sp. 2 | JR10202B | Iyale | N5 | KU317251 |
| *Sticta* sp. 2 | JR10176A | Iyale | N5 | KU317252 |
| *Sticta* sp. 2 | JR090X8b | Chawia | N5 | KU317253 |
| *Sticta* sp. 2 | JR090X8a | Chawia | N5 | KU317254 |

| | | | | |
|---|---|---|---|---|
| *Sticta* sp. 2 | JR10193C | Iyale | N5 | KU317255 |
| *Sticta* sp. 2 | JR10202C | Iyale | N5 | KU317256 |
| *Sticta* sp. 2 | UK110501a | Ngangao | N5 | KU317257 |
| *Sticta* sp. 3 | JR10K302 | Mt. Kasigau | N18 | KU317258 |
| *Sticta* sp. 3 | JR_K303 | Mt. Kasigau | P63 | KU317259 |
| *Sticta* sp. 4 | JR10194C | Iyale | N3 | KU317260 |
| *Sticta* sp. 4 | JR10194A | Iyale | N4 | KU317261 |
| *Sticta* sp. 4 | JR10194B | Iyale | N4 | KU317262 |
| *Sticta* sp. 4 | JR10212A | Iyale | N4 | KU317263 |
| *Sticta* sp. 5 | JR10117 | Vuria | P23 | KU317320 |
| *Sticta* sp. 6 | JR09D8A | Ngangao | N1 | KU317228 |
| *Sticta* sp. 6 | JR10060C | Shomoto Hill | N1 | KU317229 |
| *Sticta* sp. 7 | JR10034B | Shomoto Hill | N1 | KU317230 |
| *Sticta* sp. 7 | JR10034A | Shomoto Hill | N1 | KU317231 |
| *Sticta* sp. 7 | JR10060B | Shomoto Hill | N1 | KU317232 |
| *Sticta* sp. 7 | JR10070C | Vuria | N11 | KU317233 |
| *Sticta* sp. 7 | JR10057 | Shomoto Hill | N2 | KU317234 |
| *Sticta* sp. 7 | JR10155A | Iyale | N2 | KU317235 |
| *Sticta* sp. 7 | JR10171 | Iyale | N2 | KU317236 |
| *Sticta* sp. 7 | JR10155B | Iyale | N2 | KU317237 |
| *Sticta* sp. 7 | JR10155C | Iyale | N2 | KU317238 |
| *Sticta* sp. 7 | JR10044A | Shomoto Hill | N2 | KU317239 |
| *Sticta* sp. 7 | UK110551f | Fururu | N20 | KU317240 |
| *Sticta* sp. 7 | UK110551g | Fururu | N20 | KU317241 |
| *Sticta* sp. 7 | JR10070A | Vuria | N3 | KU317242 |
| *Sticta* sp. 7 | JR10070B | Vuria | N3 | KU317243 |
| *Sticta* sp. | JR09DA | Ngangao | N1 | KU317223 |
| *Sticta* sp. | JR10118 | Vuria | N4 | KU317224 |

**Supplementary Table 2.** Lichen species and their attributes. Prop. = proportion of; Cyanobiont diversity = Number of trnL variants / Number of specimens; d' = standardized specialization index (d'=1.00 when the taxon does not share its photobionts with other taxa); * = cephalodiate species; P = *Peltigera*-type; C = *Collema*-type; N = *Nephroma*-type; A = apothecia; S = symbiotic propagules.

| Taxon | Short code | Number of specimens | trnL type | Number of trnL variants | Reproduction method | Prop. specimens with apothecia | Prop. specimens with symbiotic propagules | Prop. shared cyanobiont variants | Cyanobiont diversity | d' |
|---|---|---|---|---|---|---|---|---|---|---|
| *Collema* sp. 1 | Co1 | 1 | P | 1 | A | 1.00 | 0.00 | 1.00 | 1.00 | 0.68 |
| *Collema* sp. 2 | Co2 | 2 | C | 2 | A | 1.00 | 0.00 | 0.00 | 1.00 | 1.00 |
| *Collema* sp. 3 | Co3 | 1 | C | 1 | S | 0.00 | 1.00 | 0.00 | 1.00 | 1.00 |
| *Collema* sp. 4 | Co4 | 2 | P | 1 | A | 1.00 | 0.00 | 1.00 | 0.50 | 0.87 |
| *Collema* sp. 5 | Co5 | 3 | C/P | 2 | A | 1.00 | 0.00 | 0.00 | 0.67 | 1.00 |
| *Collema* sp. 6 | Co6 | 1 | P | 1 | S | 0.00 | 1.00 | 0.00 | 1.00 | 1.00 |
| *Collema* sp. 7 | Co7 | 1 | P | 1 | A | 1.00 | 0.00 | 0.00 | 1.00 | 1.00 |
| *Crocodia aurata** | CrAu | 7 | P/C | 7 | S | 0.00 | 1.00 | 0.14 | 1.00 | 0.93 |
| *Crocodia clathrata** | CrCl | 1 | N | 1 | A | 1.00 | 0.00 | 1.00 | 1.00 | 0.30 |
| *Crocodia* sp. 2* | Cr2 | 7 | C/P/N | 7 | A | 1.00 | 0.00 | 0.43 | 1.00 | 0.73 |
| *Leptogium caespitosum* | LeCa | 2 | P | 2 | A | 1.00 | 0.00 | 0.50 | 1.00 | 0.83 |
| *Leptogium javanicum* | LeJa | 8 | P | 4 | A | 1.00 | 0.00 | 0.63 | 0.50 | 0.82 |
| *Leptogium juressianum* | LeJu | 5 | C | 1 | S | 0.00 | 0.80 | 1.00 | 0.20 | 0.94 |
| *Leptogium krogiae* | LeKr | 21 | C | 4 | S | 0.00 | 1.00 | 0.00 | 0.19 | 1.00 |
| *Leptogium* OTU D2 | LeD2 | 4 | C | 2 | S | 0.00 | 1.00 | 0.00 | 0.50 | 1.00 |
| *Leptogium* OTU D3 | LeD3 | 1 | C | 1 | S | 0.00 | 1.00 | 0.00 | 1.00 | 1.00 |
| *Leptogium* OTU E1 | LeE1 | 2 | P | 2 | S | 0.00 | 1.00 | 0.00 | 1.00 | 1.00 |
| *Leptogium* OTU E2 | LeE2 | 2 | C | 1 | S | 0.00 | 1.00 | 0.00 | 0.50 | 1.00 |
| *Leptogium* OTU E3 | LeE3 | 15 | P | 6 | A/S | 0.07 | 1.00 | 0.33 | 0.40 | 0.92 |
| *Leptogium* OTU F1 | LeF1 | 2 | N | 1 | S | 0.00 | 1.00 | 1.00 | 0.50 | 0.28 |
| *Leptogium* OTU H1 | LeH1 | 1 | P | 1 | A | 1.00 | 0.00 | 1.00 | 1.00 | 0.68 |
| *Leptogium* OTU H3 | LeH3 | 3 | P | 1 | S | 0.00 | 1.00 | 1.00 | 0.33 | 0.91 |
| *Leptogium* OTU J1 | LeJ1 | 15 | P/C | 7 | S | 0.00 | 1.00 | 0.40 | 0.47 | 0.92 |
| *Leptogium* OTU K1 | LeK1 | 1 | P | 1 | S | 0.00 | 1.00 | 0.00 | 1.00 | 1.00 |
| *Leptogium* OTU K3 | LeK3 | 5 | C | 2 | S | 0.00 | 1.00 | 0.00 | 0.40 | 1.00 |
| *Leptogium* OTU K4 | LeK4 | 3 | P | 1 | S | 0.00 | 1.00 | 1.00 | 0.33 | 0.91 |
| *Leptogium* OTU K5 | LeK5 | 10 | P | 2 | A | 1.00 | 0.00 | 1.00 | 0.20 | 0.80 |
| *Leptogium* OTU K6 | LeK6 | 1 | P | 1 | S | 0.00 | 1.00 | 0.00 | 1.00 | 1.00 |
| *Leptogium* OTU K7 | LeK7 | 1 | P | 1 | S | 0.00 | 1.00 | 0.00 | 1.00 | 1.00 |
| *Leptogium* OTU K8 | LeK8 | 1 | P | 1 | S | 0.00 | 1.00 | 1.00 | 1.00 | 0.60 |
| *Leptogium* OTU K10 | LeK10 | 18 | P | 6 | A/S | 0.22 | 1.00 | 0.17 | 0.33 | 0.92 |
| *Leptogium* OTU K11 | LeK11 | 2 | N | 2 | S | 0.00 | 0.50 | 1.00 | 1.00 | 0.11 |

| | | | | | | | | | | |
|---|---|---|---|---|---|---|---|---|---|---|
| *Leptogium* OTU K12 | LeK12 | 2 | P | 1 | S | 0.00 | 1.00 | 1.00 | 0.50 | 0.78 |
| *Leptogium* OTU K13 | LeK13 | 2 | N | 1 | S | 0.00 | 1.00 | 0.00 | 0.50 | 1.00 |
| *Leptogium* OTU K14 | LeK14 | 10 | N/P | 6 | A | 1.00 | 0.00 | 0.50 | 0.60 | 0.69 |
| *Leptogium* OTU L2 | LeL2 | 3 | P | 3 | A | 0.67 | 0.00 | 0.67 | 1.00 | 0.85 |
| *Leptogium* OTU L3 | LeL3 | 3 | P | 2 | S | 0.00 | 1.00 | 0.67 | 0.67 | 0.85 |
| *Leptogium* OTU L6 | LeL6 | 3 | P | 2 | S | 0.00 | 1.00 | 0.67 | 0.67 | 0.85 |
| *Leptogium* OTU O1 | LeO1 | 1 | P | 1 | A | 1.00 | 0.00 | 1.00 | 1.00 | 0.23 |
| *Leptogium* OTU P1 | LeP1 | 9 | P | 2 | S | 0.00 | 1.00 | 0.00 | 0.22 | 1.00 |
| *Leptogium* OTU Q1 | LeQ1 | 1 | P | 1 | S | 0.00 | 1.00 | 0.00 | 1.00 | 1.00 |
| *Leptogium* OTU Q2 | LeQ2 | 1 | N | 1 | A | 1.00 | 0.00 | 1.00 | 1.00 | 0.30 |
| *Leptogium* OTU R1 | LeR1 | 4 | P | 1 | S | 0.00 | 1.00 | 1.00 | 0.25 | 0.58 |
| *Leptogium* OTU R2 | LeR2 | 1 | C | 1 | S | 0.00 | 1.00 | 0.00 | 1.00 | 1.00 |
| *Leptogium* OTU R3 | LeR3 | 1 | C | 1 | S | 0.00 | 1.00 | 0.00 | 1.00 | 1.00 |
| *Leptogium* OTU R6 | LeR6 | 13 | N | 2 | S | 0.00 | 0.69 | 0.85 | 0.15 | 0.80 |
| *Leptogium* OTU R7 | LeR7 | 2 | P | 1 | A | 1.00 | 0.00 | 0.00 | 0.50 | 1.00 |
| *Leptogium* sp. (JR_W1B_R2) | - | 1 | C | 1 | - | 0.00 | 0.00 | 0.00 | 1.00 | 1.00 |
| *Leptogium* sp. (JR10251C) | - | 1 | C | 1 | S | 0.00 | 1.00 | 1.00 | 1.00 | 0.48 |
| *Leptogium* sp. (JR11094A) | - | 1 | P | 1 | S | 0.00 | 1.00 | 0.00 | 1.00 | 1.00 |
| *Lobaria retigera* | LoRe | 7 | N | 2 | S | 0.00 | 1.00 | 0.86 | 0.29 | 0.49 |
| *Lobaria* sp. 2 | Lo2 | 1 | N | 1 | S | 0.00 | 1.00 | 1.00 | 1.00 | 0.00 |
| *Pannaria* sp. 1 | Pa1 | 9 | C | 2 | S | 0.00 | 0.89 | 0.00 | 0.22 | 1.00 |
| *Pannaria* sp. 2 | Pa2 | 2 | P | 2 | S | 0.00 | 1.00 | 0.50 | 1.00 | 0.78 |
| *Pannaria* sp. 3 | Pa3 | 2 | P | 1 | A/S | 1.00 | 0.50 | 0.00 | 0.50 | 1.00 |
| *Pannaria* sp. 4 | Pa4 | 3 | P | 1 | S | 0.00 | 1.00 | 0.00 | 0.33 | 1.00 |
| *Pannaria* sp. 5 | Pa5 | 1 | P | 1 | S | 0.00 | 1.00 | 0.00 | 1.00 | 1.00 |
| *Peltigera dolichorrhiza* | PeDo | 2 | P | 1 | - | 0.00 | 0.00 | 0.00 | 0.50 | 1.00 |
| *Peltigera polydactyloides* | PePo | 2 | P | 1 | A | 0.50 | 0.00 | 1.00 | 0.50 | 0.78 |
| *Peltigera preatextata* | PePr | 2 | P | 1 | S | 0.00 | 1.00 | 1.00 | 0.50 | 0.78 |
| *Pseudocyphellaria argyraceae* | PsAr | 10 | P | 4 | S | 0.00 | 1.00 | 0.00 | 0.40 | 1.00 |
| *Pseudocyphellaria* sp. 2 | Ps2 | 17 | N | 4 | S | 0.00 | 1.00 | 0.00 | 0.24 | 1.00 |
| *Pseudocyphellaria* sp. 3 | Ps3 | 1 | N | 1 | S | 0.00 | 1.00 | 0.00 | 1.00 | 1.00 |
| *Sticta fuliginosa* | StFu | 6 | N | 2 | S | 0.00 | 1.00 | 1.00 | 0.33 | 0.61 |
| *Sticta sublimbata* | StSu | 47 | N | 6 | S | 0.00 | 0.91 | 0.87 | 0.13 | 0.75 |
| *Sticta tomentosa* | StTo | 4 | N | 1 | A | 1.00 | 0.00 | 1.00 | 0.25 | 0.66 |
| *Sticta weigelii* | StWe | 3 | N | 1 | S | 0.00 | 1.00 | 1.00 | 0.33 | 0.24 |
| *Sticta* sp. 1 | St1 | 3 | N | 1 | S | 0.00 | 1.00 | 1.00 | 0.33 | 0.58 |
| *Sticta* sp. 2 | St2 | 14 | N | 2 | S | 0.00 | 0.93 | 1.00 | 0.14 | 0.81 |
| *Sticta* sp. 3* | St3 | 2 | N/P | 2 | S | 0.00 | 1.00 | 0.00 | 1.00 | 1.00 |
| *Sticta* sp. 4 | St4 | 4 | N | 2 | S | 0.00 | 1.00 | 1.00 | 0.50 | 0.41 |
| *Sticta* sp. 5 | St5 | 1 | P | 1 | S | 0.00 | 1.00 | 1.00 | 1.00 | 0.80 |
| *Sticta* sp. 6 | St6 | 2 | N | 1 | S | 0.00 | 1.00 | 1.00 | 0.50 | 0.15 |
| *Sticta* sp. 7 | St7 | 14 | N | 5 | S | 0.00 | 1.00 | 0.86 | 0.36 | 0.43 |

**Supplementary Table 3.** Cyanobiont association patterns in the studied cyanolichen genera. C/P-type = *Collema/Peltigera*-type; *Pseudoc*. = *Pseudocyphellaria*.

| | **Collemataceae** | | | | | | **Pannariaceae** | | **Peltigeraceae** | |
|---|---|---|---|---|---|---|---|---|---|---|
| | ***Collema*** | | ***Leptogium*** | | **Family** | | ***Pannaria*** | | ***Peltigera*** | |
| | **N** | **%** | **N** | **%** | **N** | **%** | **N** | **%** | **N** | **%** |
| **Number of fungal taxa** | 7 | - | 40[a] | - | 47 | - | 5 | - | 3 | - |
| **Apotheciate taxa** | 5 | 71 | 11 | 28 | 16 | 34 | 1 | 20 | 1 | 33 |
| ***Nephroma*-type *Nostoc*** | 0 | 0 | 26 | 13 | 26 | 12 | 0 | 0 | 0 | 0 |
| **C/P-type *Nostoc*** | 11 | 100 | 180 | 87 | 191 | 88 | 17 | 100 | 6 | 100 |
| **Number of cyanobionts** | 11 | - | 206 | - | 217 | - | 17 | - | 6 | - |

| | **Lobariaceae** | | | | | | | | | |
|---|---|---|---|---|---|---|---|---|---|---|
| | ***Crocodia*** | | ***Lobaria*** | | ***Pseudoc.*** | | ***Sticta*** | | **Family** | |
| | **N** | **%** | **N** | **%** | **N** | **%** | **N** | **%** | **N** | **%** |
| **Number of fungal taxa** | 3[b] | - | 2 | - | 3[c] | - | 11[d] | - | 19 | - |
| **Apotheciate taxa** | 2 | 67 | 0 | 0 | 0 | 0 | 1 | 9 | 3 | 16 |
| ***Nephroma*-type *Nostoc*** | 4 | 27 | 8 | 100 | 18 | 64 | 100 | 98 | 130 | 85 |
| **C/P-type *Nostoc*** | 11 | 73 | 0 | 0 | 10 | 36 | 2 | 2 | 23 | 15 |
| **Number of cyanobionts** | 15 | - | 8 | - | 28 | - | 102 | - | 153 | |

[a] 35 *Leptogium* OTUs associated with C/P-type, four with *Nephroma*-type, and one with both types of *Nostoc*.

[b] One *Crocodia* species associated with C/P-type, one with *Nephroma*-type, and one with both types of *Nostoc*. All species of *Crocodia* are cephalodiate.

[c] One *Pseudocyphellaria* species associated with C/P-type and two with *Nephroma*-type *Nostoc*.

[d] One *Sticta* species associated with C/P-type, nine with *Nephroma*-type, and one cephalodiate *Sticta* species with both types of *Nostoc*.

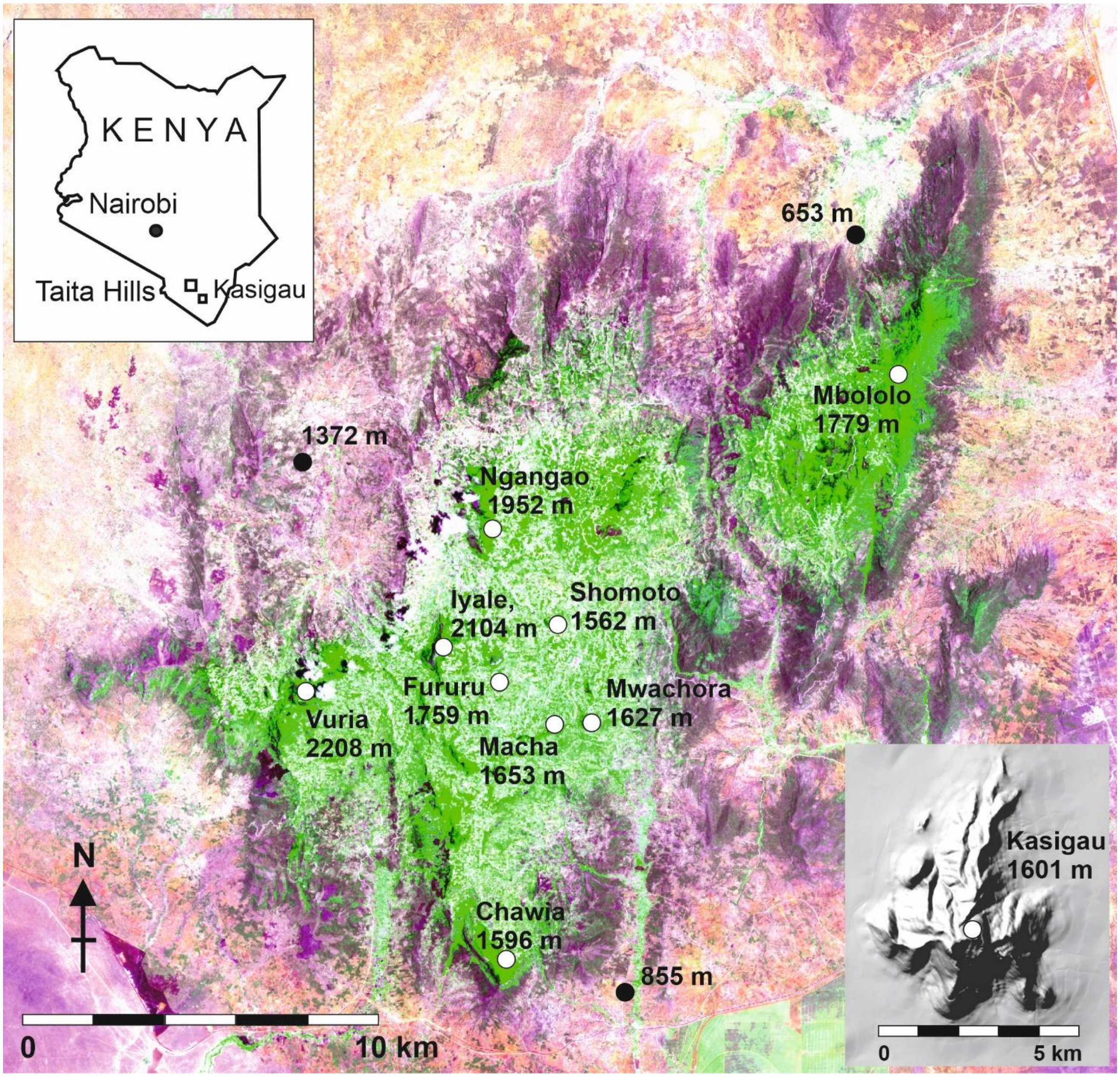


**Supplementary Figure 1.** Collection locations in the Taita Hills and Mt. Kasigau in southeastern Kenya. The sampled forest fragments (white circles) in the Taita Hills are shown on the Sentinel 2A satellite image of 2015, in which the light green areas represent agricultural areas and darker green the forests in the Taita Hills. Black dots mark elevations in the lower areas.

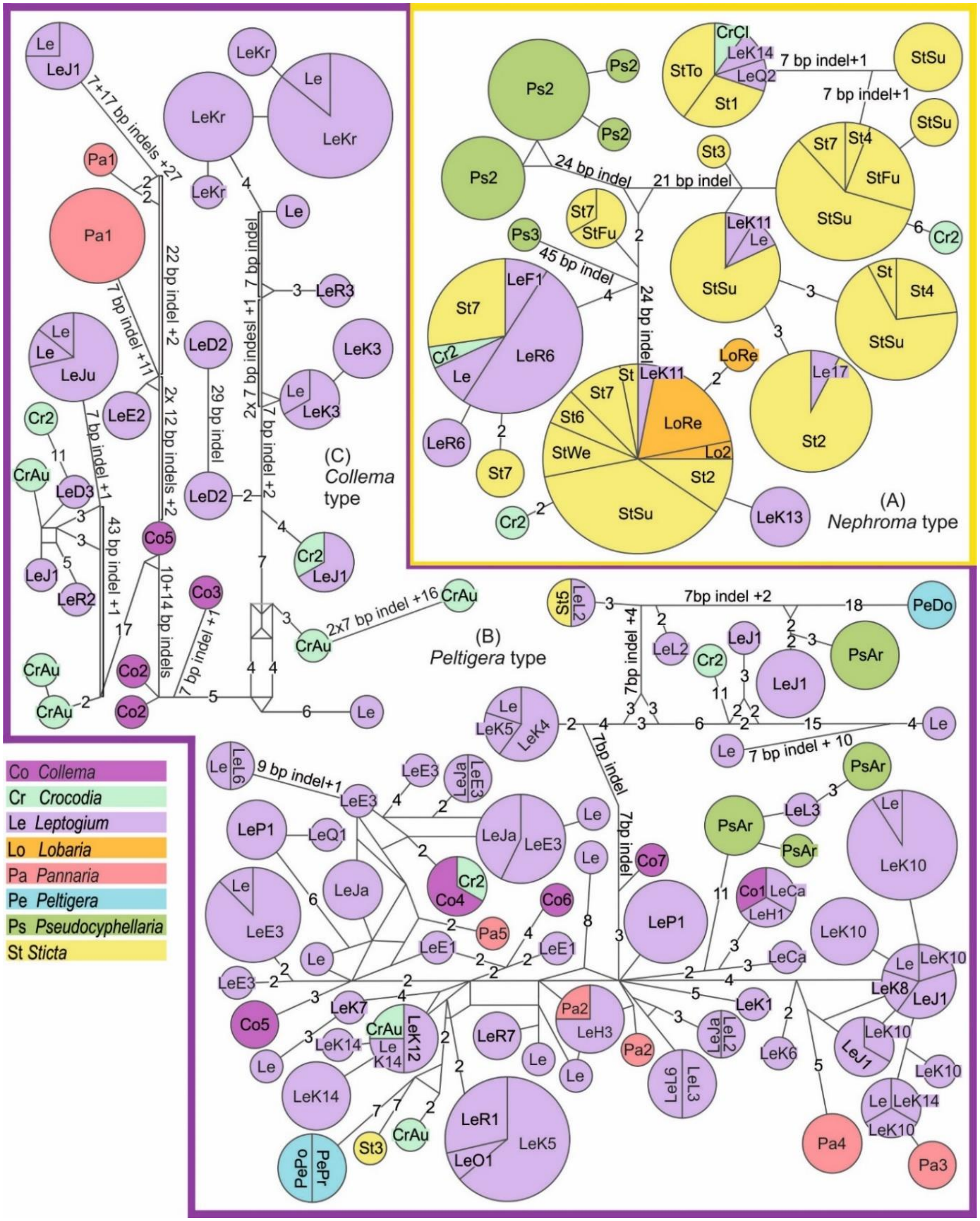


**Supplementary Figure 2.** Median-joining networks compiled of the trnL sequences of *Nephroma*-type (A), *Peltigera*-type (B), and *Collema*-type (C) *Nostoc* cyanobionts. The size of each pie chart is proportional to the number of lichen specimens from which that *Nostoc* variant was detected. The number of single nucleotide differences and indels separating the cyanobiont variants are shown on the connecting lines. The slices of the pie charts and codes refer to mycobiont taxa, colors indicating the genera; for the species matching the short codes, see Supplementary Table 2.

REFERENCES

Kaasalainen, U., Tuovinen, V., Kirika, P. M., Mollel, N. P., Hemp, A., and Rikkinen, J. (2021). Diversity of *Leptogium* (Collemataceae, Ascomycota) in East African Montane Ecosystems. *Microorganisms* 9, 314. doi: 10.3390/microorganisms9020314
